# Concurrent validity of inertial measurement units in range of motion measurements of upper extremity: A systematic review and meta-analysis

**DOI:** 10.1017/wtc.2024.6

**Published:** 2024-10-04

**Authors:** Jinfeng Li, Fanji Qiu, Liaoyan Gan, Li-Shan Chou

**Affiliations:** 1 Department of Kinesiology, Iowa State University, Ames, IA, USA; 2 Movement Biomechanics, Institute of Sport Sciences, Humboldt-Universität zu Berlin, Berlin, Germany; 3 Faculty of Kinesiology, Sport, and Recreation, College of Health Science, University of Alberta, Edmonton, AB, Canada

**Keywords:** Inertial Measurement Unit (IMU), Movement Analysis, Upper Extremity

## Abstract

Inertial measurement units (IMUs) have proven to be valuable tools in measuring the range of motion (RoM) of human upper limb joints. Although several studies have reported on the validity of IMUs compared to the gold standard (optical motion capture system, OMC), a quantitative summary of the accuracy of IMUs in measuring RoM of upper limb joints is still lacking. Thus, the primary objective of this systematic review and meta-analysis was to determine the concurrent validity of IMUs for measuring RoM of the upper extremity in adults. Fifty-one articles were included in the systematic review, and data from 16 were pooled for meta-analysis. Concurrent validity is excellent for shoulder flexion–extension (Pearson’s *r* = 0.969 [0.935, 0.986], ICC = 0.935 [0.749, 0.984], mean difference = −3.19 (*p* = 0.55)), elbow flexion–extension (Pearson’s *r* = 0.954 [0.929, 0.970], ICC = 0.929 [0.814, 0.974], mean difference = 10.61 (*p* = 0.36)), wrist flexion–extension (Pearson’s *r* = 0.974 [0.945, 0.988], mean difference = −4.20 (*p* = 0.58)), good to excellent for shoulder abduction–adduction (Pearson’s *r* = 0.919 [0.848, 0.957], ICC = 0.840 [0.430, 0.963], mean difference = −7.10 (*p* = 0.50)), and elbow pronation–supination (Pearson’s *r* = 0.966 [0.939, 0.981], ICC = 0.821 [0.696, 0.900]). There are some inconsistent results for shoulder internal–external rotation (Pearson’s *r* = 0.939 [0.894, 0.965], mean difference = −9.13 (*p* < 0.0001)). In conclusion, the results support IMU as a viable instrument for measuring RoM of upper extremity, but for some specific joint movements, such as shoulder rotation and wrist ulnar-radial deviation, IMU measurements need to be used with caution.

## 1. Background

Range of motion (RoM) describes the extent of movement achievable around a joint or at a specific point of the body. Measuring RoM is essential in clinical assessments, such as in evaluating shoulder joint mobility for the diagnosis and staging of frozen shoulder (Ješić et al., 2022). Accurate and reliable RoM measurements are critical for clinicians in guiding their diagnostic and treatment strategies. In clinical settings, goniometers have become a popular choice for RoM measurement due to their affordability, portability, and user-friendly nature. Nonetheless, they have notable limitations. First, goniometers can only measure joint angles in a single plane and static positions, which restricts their ability to assess dynamic joint movements. Second, the reliability and accuracy of measurements taken with goniometers can vary widely. The intraclass correlation coefficients (ICCs) for RoM measurements in shoulder and elbow joints range from 0.76 to 0.94 and 0.36 to 0.91 (Muir et al., 2010; Walmsley et al., 2018), respectively. Such variability in measurement reliability may stem from the anatomical specificity of the joints being measured and the different levels of experience among evaluators. Consequently, goniometers should be considered as a basic tool for RoM measurement. Their substantial measurement errors limit their utility in more precise clinical research and kinematic studies.

Commercial marker-based motion capture systems, also known as optical motion capture systems (OMCs), such as Vicon (Vicon Motion Systems Ltd., Oxford, UK), are widely regarded as the “gold standard” in clinical human motion analysis and biomechanics research (Nagymáté and Kiss, 2018; Valevicius et al., 2018), with a systematic review noting within-assessor errors less than 4.0° in the sagittal plane and below 2.0° in the frontal plane for gait measurements (McGinley et al., 2009). For such systems, passive reflective markers are strategically placed on specific bony landmarks of the body, corresponding to the segments to be analyzed. These markers reflect light back to cameras, enabling the associated biomechanics model to reconstruct three-dimensional human motion in space (Colyer et al., 2018). However, these systems come with considerable limitations: costly, lack portability, necessitate a dedicated laboratory setting, and involve lengthy setup and calibration procedures (Sessa et al., 2013; Wu et al., 2022), making these systems impractical for routine clinical use. Furthermore, the occlusion of markers by clothing can significantly affect the reliability of results, limiting the marker-based system’s application in real-world scenarios (van der Kruk and Reijne, 2018).

Inertial measurement units (IMUs), or wearable sensors, have emerged as an alternative method that can overcome these limitations. IMUs are widely used in human kinematics analysis research and clinical gait assessments due to their portability and affordability. Typically, IMUs consist of three-axis accelerometers, three-axis gyroscopes, with or without three-axis magnetometers (Seel et al., 2014). Users can estimate the kinematic parameters of body segments in three-dimensional space through data fusion algorithms and biomechanics models (Poitras et al., 2019). Therefore, the use of multiple IMUs can provide the possibility of collecting upper limb motion parameters in daily life. While IMUs show promise as tools for motion tracking, it is essential to conduct thorough metrological validation to ensure their validity and reliability before they can be adopted for widespread use. Many studies have examined their validity and reliability in measuring human kinematic parameters during various movements. Several systematic reviews and meta-analyses on the validity and reliability of IMU measurement of lower limb kinematics exist (Kobsar et al., 2020; Zeng et al., 2022), the results demonstrated that IMUs are reliable tools for measuring the RoM in the lower limbs. In the context of upper extremities, a systematic review by Walmsley et al. (2018) analyzed 22 studies conducted before 2018, found that while IMUs exhibited higher error margins in vivo compared to OMC systems, achieving errors less than 5° was possible with significant customization. Furthermore, another systematic review highlighted the broad error margins of IMUs in measuring upper limb joint motions: for shoulder joints (root mean square error [RMSE] 0.2°–64.5°), elbow joints (RMSE 0.2°–30.6°), and wrists (RMSE 2.2°–30°) (Poitras et al., 2019), when compared to OMC systems. However, there is a notable gap in the literature regarding meta-analysis on the validity of IMU measurements in upper extremity motion analysis. While some articles have systematically reviewed the measurement validity of IMUs for upper limb joint motion, they predominantly provide qualitative summaries. There is a lack of high-quality meta-analysis that quantitatively assesses the measurement validity of IMUs and examines the statistical significance of the measurement errors. Therefore, it is necessary to quantitatively validate IMU systems before using them routinely in assessments. The main objectives of this review study were: (1) to provide a summary of the characteristics of commercially available wearable sensors, (2) to quantitatively summarize the existing psychometric properties by comparing IMUs with OMCs, and (3) to establish evidence supporting the use of IMUs for measuring RoM in the upper limb.

## 2. Methods

### 2.1. Protocol and registration

This systematic review and meta-analysis adhered to the Preferred Reporting Items for Systematic Reviews and Meta-Analyses (PRISMA) guidelines (Page et al., 2021), and the protocol was registered on the International Prospective Register of Systematic Review on December 28, 2022 (PROSPERO number: CRD42022384738).

### 2.2. Searching strategy

We selected relevant studies published between January 1, 2016 and December 19, 2022, by searching PubMed, Web of Science, Scopus, IEEE Xplore electronic databases, and ClinicalTrials register system. The search terms included wearable sensor, motion analysis, range of motion, upper limbs, and optical motion capture system. The specific search strategies in PubMed included: (wearable sens*[Title/Abstract] OR inertial motion unit*[Title/Abstract] OR inertial movement unit*[Title/Abstract] OR inertial sens*[Title/Abstract] OR sensor[Title/Abstract] OR accelerometer*[Title/Abstract] OR gyroscope*[Title/Abstract]) AND (movement*analysis[Title/Abstract] OR motion analysis*[Title/Abstract] OR motion track*[Title/Abstract] OR track* motion*[Title/Abstract] OR measurement system*[Title/Abstract] OR movement[Title/Abstract]) AND (joint angle*[Title/Abstract] OR angle*[Title/Abstract] OR kinematic*[Title/Abstract] OR range of motion*[Title/Abstract]) AND (upper limb*[Title/Abstract] OR upper extremit*[Title/Abstract] OR arm*[Title/Abstract] OR elbow*[Title/Abstract] OR wrist*[Title/Abstract] OR shoulder*[Title/Abstract] OR humerus*[Title/Abstract]) AND (motion capture system[Title/Abstract] OR 3D motion capture[Title/Abstract] OR marker*[Title/Abstract] OR optical[Title/Abstract] OR camera*[Title/Abstract] OR optoelectronic[Title/Abstract]) NOT (review[Title/Abstract]) AND (Filter: 2016–2022). The basic search terms are similar for different databases with very minor adjustments. In addition, we performed a manual search using the references of previous review articles. Complete search strategy for all databases can be seen in Table 1.

**Table 1.** Complete search strategy

Search strategy in PubMed	Search strategy in Web of Science
(((((((((wearable sens*[Title/Abstract]) OR (inertial motion unit*[Title/Abstract])) OR (inertial movement unit*[Title/Abstract])) OR (inertial sens*[Title/Abstract])) OR (sensor[Title/Abstract])) OR (accelerometer*[Title/Abstract])) OR (gyroscope*[Title/Abstract])) AND ((((((movement* analysis[Title/Abstract]) OR (motion analysis*[Title/Abstract])) OR (motion track*[Title/Abstract])) OR (track* motion*[Title/Abstract])) OR (measurement system*[Title/Abstract])) OR (movement[Title/Abstract]))) AND ((((joint angle*[Title/Abstract]) OR (angle*[Title/Abstract])) OR (kinematic*[Title/Abstract])) OR (range of motion*[Title/Abstract]))) AND (((((((upper limb*[Title/Abstract]) OR (upper extremit*[Title/Abstract])) OR (arm*[Title/Abstract])) OR (elbow*[Title/Abstract])) OR (wrist*[Title/Abstract])) OR (shoulder*[Title/Abstract])) OR (humerus*[Title/Abstract]))) AND (((((motion capture system[Title/Abstract]) OR (3D motion capture[Title/Abstract])) OR (marker*[Title/Abstract])) OR (optical[Title/Abstract])) OR (camera*[Title/Abstract])) OR (optoelectronic[Title/Abstract])) NOT (review[Title/Abstract]) AND (Filter:2016–2022)	((TS = (wearable sens* OR inertial motion unit* OR inertial movement unit* OR inertial sens* OR sensor) AND (TS = (movement* analysis OR motion analysis* OR motion track* OR track* motion* OR measurement system OR movement)) AND (TS = (motion capture system OR 3D motion capture OR marker* OR optical OR camera*)) AND (TS = (joint angle* OR angle* OR kinematic* OR range of motion*)) AND (TS = (upper limb* OR upper extremit* OR arm* OR elbow* OR wrist* OR shoulder* OR humerus*))))
Search strategy in Scopus	Search strategy in IEEE
(TITLE–ABS–KEY (wearable AND sens* OR inertial AND motion AND unit* OR inertial AND movement AND unit* OR inertial AND sens* OR sensor)) AND (TITLE–ABS–KEY (movement* AND analysis OR motion AND analysis* OR motion AND track* OR track* AND motion* OR measurement AND system* OR movement)) AND (TITLE–ABS–KEY (joint AND angle* OR angle* OR kinematic* OR range AND of AND motion*)) AND (TITLE–ABS–KEY (upper AND limb* OR upper AND extremit* OR arm* OR elbow* OR wrist* OR shoulder* OR humerus*)) AND (TITLE–ABS–KEY (motion AND capture AND system OR 3D AND motion AND capture OR marker* OR optical OR camera*))	(“All Metadata”:wearable sens* OR “All Metadata”:inertial motion unit* OR “All Metadata”:inertial movement unit* OR “All Metadata”:inertial sens* OR “All Metadata”:sensor) AND (“All Metadata”:motion capture system OR “All Metadata”:3D motion capture OR “All Metadata”:marker* OR “All Metadata”:optical OR “All Metadata”:camera*) AND (“All Metadata”:upper limb* OR “All Metadata”:upper extremit* OR “All Metadata”:arm* OR “All Metadata”:elbow* OR “All Metadata”:wrist* OR “All Metadata”:shoulder* OR “All Metadata”:humerus*) AND (“All Metadata”:joint angle* OR “All Metadata”:angle* OR “All Metadata”:kinematic* OR “All Metadata”:range of motion*) AND (“All Metadata”:movement* analysis OR “All Metadata”:motion analysis* OR “All Metadata”:motion track* OR “All Metadata”:track* motion* OR “All Metadata”:measurement system* OR “All Metadata”:movement)

* (Asterisk) is to replace any number of character, for example, extremit* finds “extremity” and “extremities.”

### 2.3. Inclusion and exclusion criteria

Articles that met the following criteria were included in this systematic review: (1) evaluated the validity of IMUs, (2) measured and reported specific upper extremity RoM results, (3) compared the measurements captured by IMUs to the marker-based motion capture systems, (4) assessed human beings, (5) published in English. The exclusion criteria were as follows: (1) no relevant outcomes, (2) no comparison with standard marker-based motion capture systems, (3) only assessed lower limb motion, (4) only assessed unnatural human motion, (5) animal model studies, (6) only assessed children and infants, (7) no research studies or no full text, (8) published in other languages. Additional details on inclusion and exclusion criteria can be seen in Supplementary file 1.

### 2.4. Study selection

All the results were entered into the bibliographic management tool (Endnote X9, Thomson Reuters, New York, USA), and duplicates were removed by Endnote automatically. Two authors (Li and Qiu) independently screened the titles and abstracts retrieved to include the articles that satisfied the criteria, and then read the full texts for final eligibility. Any disagreements were resolved by consensus with the third reviewer (Gan).

### 2.5. Assessment of risk of bias and level of evidence

The included studies were assessed according to the Critical Appraisal of Study Design for Psychometric Articles. To make it more appropriate for assessing the psychometrics of IMUs, Kobsar et al. (2020) modified the checklist. This modified evaluation checklist contains 12 items in five different domains: (1) study question, (2) study design, (3) measurements, (4) analyses, and (5) recommendations. Each item is rated as 0, 1, or 2, with a maximum total score of 24. The specific scoring criteria and descriptors for each item can be found in Supplementary file 2. It should be noted that the question #6 pertains solely to literature that covers reliability testing in methodology (i.e., patient reevaluation), but not all the literature included in the review needs to be assessed for this specific question. Therefore, total score for literature that is not relevant to this question item is 22 points. Initially, two reviewers (Li and Qiu) evaluated three articles simultaneously, discussing and reaching consensus on each item, and then two reviewers used the same criteria to evaluate the remaining literature separately. The agreement between the two raters was assessed using Cohen’s kappa coefficient. An acceptable level of agreement is typically indicated by a Cohen’s kappa coefficient greater than 0.60 (Henry et al., 2016). Two raters discussed and resolved most disagreements, and if consensus could not be reached, a third rater (Gan) was invited to adjudicate.

According to the score percentage, the quality of the included literature can be divided into four categories (Kobsar et al., 2020): (1) score percentage greater than 85% were classified as high quality (HQ), (2) between 70 and 85% were classified as moderate quality (MQ), (3) between 50 and 70% were classified as low quality (LQ), (4) below 50% were considered very low quality (VLQ). The results of quality assessment were then used in determining the level of evidence (van Tulder et al., 2003):

1. Strong: Consistent results among multiple HQ studies.
2. Moderate: Consistent results among multiple MQ studies and/or only one HQ study.
3. Limited: Consistent results among multiple LQ studies and/or only one MQ study.
4. Very limited: Consistent results among multiple VLQ studies and/or only one LQ study.
5. Conflicting: Inconsistent results among multiple trials, regardless of study quality.

### 2.6. Data extraction

Data extraction and results compilation were performed by two independent reviewers (Li and Qiu) and data were extracted into Microsoft Excel. In case of disagreement, a third researcher (Gan) intervened. Data extracted from the studies included the following information: (1) study information (author and publication year), (2) sample size, (3) wearable sensor information (sensor brand, sampling rate, data fusion algorithm/filter, calibration methods, and placement), (4) reference system, (5) measured joints and/or movements (shoulder, elbow, and wrist, or specific complex movement), and (6) results and statistical parameters.

Since this meta-analysis only considers the validity of the IMU for measuring the RoM of the upper extremity compared with the marker-based motion capture system, the extracted statistics include: mean ± standard deviation (SD), ICC, Pearson’s *r*, RMSE, bias (mean difference), limits of agreement (LoA), and other statistics (i.e., coefficient of determination [*r* ^2^] and coefficient of multiple correlation [CMC]). Then, according to different upper limb joints and joint motion planes, the extracted data were divided into the following groups: shoulders (flexion/extension, abduction/adduction, and internal/external rotation), elbows/forearms (flexion/extension and pronation/supination), and wrists (flexion/extension and ulnar/radial deviation).

### 2.7. Statistical analysis

The meta-analysis was performed using the Review Manager version 5.4.1 (The Cochrane Collaboration, Copenhagen, Denmark). Data for validity outcomes were meta-analyzed based on the mean ± SD, ICC, and Pearson’s *r.* The agreement metrics of ICCs were interpreted as (Han, 2020): (1) poor (< 0.500), (2) moderate (0.500–0.749), (3) good (0.750–0.899), and (4) excellent (≥ 0.900), and *r* was interpreted as (Wahyuni and Purwanto, 2020): (1) very weak relationship (< 0.2), (2) weak relationship (0.2–0.4), (3) moderate relationship (0.4–0.6), (4) strong relationship (0.6–0.8), and (5) very strong relationship (>0.8). Point estimates were weighted based on the sample size of the included studies and considering the non-normality of the two parameters (ICC and *r*). It was necessary to perform Fisher’s *Z*-transformation (Cozzolino, 2009; Kobsar et al., 2020) and then transformed back to ICC/*r* for reporting, the formula are as follows (Kobsar et al., 2020; Zeng et al., 2022):

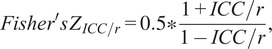




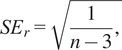




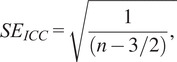




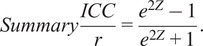



Sensitivity analysis was considered when there was heterogeneity among the studies and the number of studies was greater than or equal to three. Heterogeneity in the data was assessed using Tau^2^ and *I* ^2^ statistics. A Tau^2^ value of 0 indicates an absence of heterogeneity. *I* ^2^ values are interpreted as follows: less than 25% indicates low heterogeneity, 26–50% suggests moderate heterogeneity, and over 75% points to high heterogeneity (Higgins et al., 2003; Walmsley et al., 2018; Zeng et al., 2022). When the *I* ^2^ value exceeded 50%, a sensitivity analysis was conducted. This involves sequentially excluding each included study and then performing a meta-analysis on the remaining studies. If, after exclusion, the *I* ^2^ decreases to below 50% and the meta-analysis results remain unchanged, it indicates robustness in the original meta-analysis findings. Conversely, if the *I* ^2^ decreases below 50% but the results of the meta-analysis change, it suggests non-robustness in the original meta-analysis outcomes. The level of significance was *p* < 0.05. Given the heterogeneity of the trial conditions of the included studies, a random effects model was used with 95% CI (Huedo-Medina et al., 2006). When the number of studies was sufficient (*n* ≥ 3) (Zeng et al., 2022), subgroup analyses were conducted to explore possible associations between the study characteristics and the validity of the IMUs measurements.

## 3. Results

### 3.1. Characteristics of the included studies

**Figure 1.**
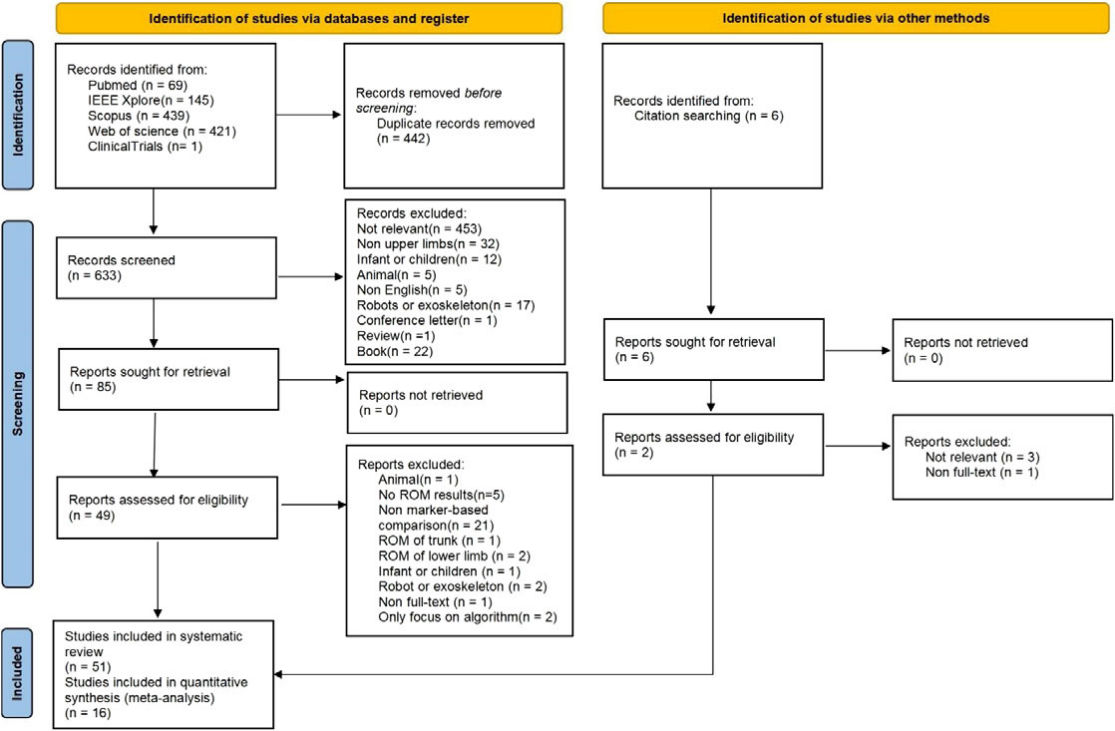
Study selection according to PRISMA flow diagram 2020.

Our search strategy identified a total of 1,081 articles through databases and cross-referencing. Following the removal of duplicates, 639 articles remained. After screening titles, abstracts and full-text, 51 articles were included in this systematic review. A PRISMA flow chart showing the screening process is presented in Figure 1. Data from 491 adults were included across these studies (sample size: 9.6 (5.8); median sample size: 10; range: 1–24). The most common sampling frequency for wearable sensors was 100 Hz (*n* = 15, range: 20–1,000 Hz). All the characteristics of the included articles are shown in Table 2.

**Table 2.** Basic information of included studies

Study information		Sample size (*N*)	Sensor brands	Sampling rate of sensors (Hz)	Data fusion algorithm/filter	Placement of sensors (only upper limbs)	Calibration methods	Marker-based motion capture system	Measured joints and/or movements			
Author	Year								Shoulder	Elbow/forearm	Wrist	Specific tasks
Abbasi–Kesbi et al.	2018	1	MPU–9150 (InvenSense)	50	Complementary filter + L2 norm total variation algorithm	S1: wrist S2: elbow S3: shoulder (reference)	N/A	Qualisys	N/A	Flex./Ext.	Flex/Ext.	Movement 1: Changes the joint angle of his wrist and elbow simultaneously 90 degrees from the first state counterclockwise and then returns them to the first state Movement 2: Angular variations simultaneously until reaching angular variations of wrist to 150 degrees and elbow to 90 degrees
Alarcon–Aldana et al.	2022	1	Customized (Imocap–GIS)	60 (default)	Kalman filter + Madwik filters	S1: arm (outer side in the lower third of the arm segment) S2: forearm (the lower third of the forearm)	Static calibration	OptiTrack	N/A	Flex./Ext. Prona‥/Supin.	N/A	N/A
Barreto et al.	2021	10	Xsens	240	N/A	S1: shoulders (middle of the scapula spine) S2: upper arms (lateral side above elbow) S3: forearms (medial side of wrist) S4: hands (posterior side)	N–pose + walk + N–pose	Qualisys	Abd./Add. Flex./Ext. IR/ER	Flex./Ext.	Flex./Ext.	Round–off back handsprings
Bessone et al.	2022	14	Aktos–t system	143	10 Hz, 2nd Butterworth filter	S1: upper arms S2: forearm S3: hands	T–pose	Vicon	Flex./Ext. Abd./Add.	Flex./Ext.	Flex./Ext. Ulnar deviation	1. Repetitive movement test 2. Walking 3. Squat jumping
Boddy et al.	2019	10	MotusBASEBALL	1,000	N/A	S: forearm (2 finger widths below the medial epicondyle)	N/A	OptiTrack	Rotation	N/A	N/A	Pitches of baseball
Callejas–Cuervo et al.	2017	1	Customized (MPU–9150)	30	Kalman filter	S1: upper arm (external part of humerus) S2: forearm (between radial styloid and ulnar styloid)	N/A	Qualisys	N/A	Flex./Ext.	N/A	N/A
Camp et al.	2021	10	MotusBASEBALL	1,000	N/A	S: forearm (2 finger widths below the medial epicondyle)	N/A	Raptor	Rotation	N/A	N/A	Pitches of baseball
Chan et al.	2022	19	XCLR8 IMU	20	N/A	S1: forearm	N/A	Motion Analysis Corp.	Flex./Ext. ER. Abd.	N/A	N/A	N/A
Chapman et al.	2019	10	APDM	128	5 Hz, 5th Butterworth filter	S1: humerus S2: sternum (reference)	N/A	OptiTrack	Flex. Abd. IR/ER	N/A	N/A	N/A
Chen et al.	2020	14	APDM	128	Kalman filter versus complementary filter	S1: upper arm S2: forearm S3: hand	Stationary calibration	OptiTrack	N/A	N/A	N/A	1. Grasping a wooden dowel 2. Transferring the dowel to the unloading container 3. Returning the hand back to the material feed container to grasp the next dowel
Choo et al.	2022	7	PNS (Perception Neuron)	120	N/A	S1: upper arms S2: wrists S3: hands	Perception Neuron 4–step calibration	Vicon	Flex./Ext. Abd./Add.	Flex./Ext.	N/A	1. Stationary walk 2. Distance walk 3. Stationary jog 4. Distance jog 5. Stationary floorball wrist shot 6. Moving wrist shot
Contreras–Gonzalez et al.	2020	9	LP–RESEARCH (LPMS–URS2)	400	Kalman filter	S1: shoulder (shoulder ends) S2: elbow (beginning of elbow)	Functional calibration	OptiTrack	Abd./Add. Flex./Ext. Horiz. Add. Rotation	N/A	N/A	N/A
Digo et al.	2022	6	Xsens	50	Gradient descent algorithm	S1: right upper arm S2: right forearm S3: thorax	A algebraic quaternion	OptiTrack	Elevation	Flex./Ext.	N/A	Place the box over the cross marked on the table
Dufour et al.	2021	11	Xsens	100	N/A	S1: chest S2: upper arm	N/A	OptiTrack	Elevation	N/A	N/A	place a small machine screw in a small bin 0.56 m in front of them using their right hand
Rovini et al.	2019	20	SensHand (DAPHNE)	100	Custom–made algorithms	S1: forearm	N/A	SMART DX	N/A	N/A	N/A	Forearm pronation–supination
Ertzgaard et al.	2016	10	Customized	100	Kalman filter	S1: upper body S2: upper arms S3: lower arms	Functional calibration	CodaMotion	Flex./Ext. Abd./Add. ER/IR	Flex. Prona./Supina.	N/A	1. A lifting and dropping task, where the subject moved 4 cones from one lower level on a table to a higher in a forward direction 2. A throwing and catching task that mainly involved elbow flexion 3. Hands moved from the starting position to the top of the head, to the shoulder, clapping back of hands together, moved hands to the knee and then to the toe 4. The hands moved from the starting position to the ears, to the eyes and then to the mouth
Esfahani et al.	2018	1	Customized	100	Kalman filter with adaptive approach	S1: hands S2: forearms S3: upper arms S4: sternum	Accelerometer calibration + gyroscope calibration	Vicon	Flex.(45,90,135,180) Ext. Abd.(45,90,135,180) IR/ER	N/A	N/A	N/A
Fantozzi et al.	2016	8	APDM	128	Kalman–based algorithm	S1: sternum S2: humerus S3: forearm (just above the ulnar and radial styloids) S4: hand	Static calibration + functional calibration	SMART DX	Flex./Ext. Abd./Add. IR/ER	Flex./Ext. Prona./Supina.	Flex./Ext. Radial–ulnar deviation	Front–crawl swimming Breaststroke swimming
Fischer et al.	2021	10	IMU DyCare	104	N/A	S1: hand S2: forearm	N/A	Vicon	N/A	N/A	Flex./Ext. Radial–ulnar angle	Dart–throwing motion task
Goreham et al.	2022	15	Notch	40	N/A	S1: sternum S2: forearms (50% of distance between distal and proximal segment landmarks) S3: upper arms	Static and dynamic calibration	OptiTrack	Flex./Ext. Abd./Add. IR/ER	Flex./Ext.	N/A	1. Hand to back pocket 2. Hand to the contralateral shoulder 3. Hand to the top of head
Guignard et al.	2021	5	Hikob Fox	100	6 Hz, 4th Butterworth filter	S1: upper arm S2: forearm (50% of distance between distal and proximal segment landmarks)	Functional calibration	OptiTrack	N/A	Flex./Ext. Prona./Supina.	N/A	1. Walking 2. Crawl 3. Rowing
Henschke et al.	2022	24	Wave Track	147	N/A	S1: sternum (10 cm below the jugular fossa) S2: upper arm (15 cm below the acromion process)	T–pose	Vicon	Abd./Add. ER/IR Vertical Flex./Ext. Horiz. Flex./Ext.	N/A	N/A	Multiplanar shoulder–elbow movement
Hubaut et al.	2022	10	Dramco KUL	50	6 Hz, 4th Butterworth filter	S1: center of forearm S2: center of upper arm S3:C7 vertebra	N/A	Vicon	N/A	N/A	N/A	1. Pushing the pole 2. Snatching the mass with the pole 3. Pulling the pole
Humadi et al.	2021	10	Xsens	60	3rd one–dimensional median filter	S1: upper arms S2: forearms S3: hands	N–pose	Vicon	Add. Flex. Rotation	Flex./Ext.	N/A	1. Packing 2. Package inspection 3. Reaching an object
Laidig et al.	2017	1	Xsens	N/A	N/A	S1: upper arm close to the elbow S2: forearm close to the wrist	Anatomical calibration	Vicon	N/A	Flex./Ext. Prona./Supin.	N/A	N/A
Ligorio et al.	2017	15	YEI technology	220	Kalman filter	S1: upper arm close to the elbow S2: forearm close to the wrist	Functional calibration + static N–pose	Vicon	N/A	Flex./Ext. Prona./Supina.	N/A	N/A
Ligorio et al.	2020	10	Pivot	100	Kalman filter	S1: hand S2: forearm S3: upper arm S4: sternum	N–pose	Vicon	Flex./Ext. Abd./Add. IR.ER	Flex./Ext. Carrying angle Prona./Supina.	Flex.Ext. Abd./Add. IR/ER	Sun salutation
Lin et al.	2021	13	Customized (WISN)	75	Quaternion–based complementary nonlinear filter	S1: sternum (xiphoid process) S2: upper arm (middle point between the acromion and lateral epicondyle of the humerus) S3: forearm (middle point between the olecranon process and the styloid process of the ulna)	N/A	Vicon	Flex./Ext. Abd. ER/IR	N/A	N/A	N/A
Marta et al.	2020	2	Customized	20	Kalman filter	S1: trunk S2: upper arm S3: low arm	Static + dynamic calibration	Smart DX	Flex./Ext. Abd./Add.	N/A	N/A	N/A
Mavor et al.	2020	20	Xsens	240	N/A	S1: sternum S2: upper arms S3: forearms S4: hands	N/A	Vicon	Flex./Ext. Abd./Add. Axial Rotation	Flex./Ext. Abd./Add. Axial Rotation	N/A	1. Kneel–to–prone 2. Kneel–to–run 3. Prone–to–run 4. Walking 5. Prone–to–kneel 6. Run–to–kneel 7. Run–to–prone 8. Running
Mihcin,et al.	2019	1	Smartsuit Pro	100	N/A	S1: hands S2: forearms S3: upper arms S4: shoulders	N/A	OptiTrack	Flex.	Flex.	N/A	The shoulder and also elbow flexion movements both begin with the palms against subject’s sides of a body. Then the subject raises the right/left arm in front of the body to highest point over the head
Morrow et al.	2017	6	APDM	80	6 Hz, 4th Butterworth filter	S1: sternum S2: upper arm S3: forearm	Static calibration	Motion Analysis Corp.	Elevation	Flex.	N/A	Surgical task
Muller et al.	2017	1	Xsens	N/A	Gradient descent algorithm	S1: upper arm S2: wrist	N/A	Vicon	N/A	Flex./Ext. Prona./Supina.	N/A	Grabbing a door knob, turning the knob and opening the door
Ohberg et al.	2019	20	MoLab POSE	128	N/A	S1: xiphoid process S2: upper arm S3: forearm	N/A	Qualisys	Flex./Ext. Abd./Add. IR/ER	Flex./Ext. IR/ER	N/A	1. Drinking task 2. Finger to nose task
Pedro et al.	2021	18	Xsens	N/A	N/A	S1: sternum S2: upper arms S3: forearms (close to wrist) S4: hands	N/A	Qualisys	Flex./Ext. Abd./Add. IR/ER	Flex./Ext. Prona./Supina.	Flex./Ext.	Hitting the ball as fast as they can against a hanging cotton cloth of 3X2 to cushion the ball
Picerno et al.	2019	14	Xsens	100	N/A	S1: thorax S2: upper arm S3: forearm	Anatomical calibration	Smat DX	Elevation Axial Rotation	Flex./Ext. Prono./Supina.	N/A	N/A
Poitras et al.	2019	16	Xsens	60	N/A	S1: shoulders S2: sternum S3: upper arms S4: forearms	N–pose	Vicon	Flex. Abd.	N/A	N/A	Lifting tasks
Robert–Lachaine et al.	2020	5	Perception Neuron	120	Dynamic time wrapping algorithm	S1: sternum S2: upper arms S3: forearms	Steady pose + A–pose + T–pose + S–pose	Optotrak	Flex./Ext. Abd./Add. IR/ER	Flex./Ext. Prona./Supina.	Flex./Ext.	Moved five empty boxes one at the time from four stations at each corner of the platform
Robert–Lachaine et al.	2017b	12	Xsens	30	N/A	S1: sternum S2: upper arms S3: forearms S4: hands	T–pose	Optotrak	Flex./Ext. Abd./Add. IR/ER	Flex./Ext. Prona./Supina.	Flex./Ext.	Moved five empty boxes one at the time from four stations at each corner of the platform
Robert–Lachaine et al.	2017a	12	Xsens	30	N/A	S1: sternum S2: upper arms S3: forearms S4: hands	T–pose + N–pose	Optotrak	N/A	N/A	N/A	1.Turning gait 2.Deep squat to pick a box
Ruiz–Malagon et al.	2022	15	NOTCH	100/250/500	N/A	S1: upper arm S2: forearm	Steady pose	Qualisys	N/A	Flex./Ext. Prona./Supina.	N/A	Hitting ball
Schall et al.	2016	6	I2 M Motion Tracking	20	Complementary weighting algorithm	S1: sternal notch S2: upper arm	N/A	Vicon	Elevation	N/A	N/A	Milking cluster attachment task
Sers et al.	2020	8	Perception Neuron	120	N/A	S1: upper spine (C7) S2: center of humerus S3: acromions	Perception Neuron 4–step calibration	Vicon	Abd.	N/A	N/A	N/A
Shepherd et al.	2017	4	SABELSense	100	Heading Reference System orientation filter	S1: superior to the distal radioulnar articulation	T–pose	Vicon	Flex./Ext. Abd./Add/	Flex./Ext.	Flex./Ext.	Synchronized ball release
Slade et al.	2022	5	OpenSenseRT	30	Mahony filter	S1: upper arms S2: forearms S3: hands S4: torso	Static calibration	OptiTrack	Flex Add, Rotation	Flex.	Flex.	1. Pick up a knife from table 2. Cutting 3. Place the knife back on table
Truppa et al.	2021a	10	TuringSense	100	Masgwick filter versus Mahony filter versus Customized filter	S1: hand S2: forearm S3: upper arm S4: trunk	N/A	Vicon	Flex./Ext. Abd./Add. IR.ER	Flex./Ext. Carrying angle Prona./Supina.	Flex.Ext. Abd./Add. IR/ER	Sun salutation
Truppa et al.	2021b	5	TuringSense	100	Extend Kalman filter	S1: hand S2: forearm S3: upper arm S4: trunk	N–pose	Vicon	Flex./Ext. Abd./Add. IR.ER	Flex./Ext. Carrying angle Prona./Supina.	Flex.Ext. Abd./Add. IR/ER	Sun salutation
Truppa et al.	2022	10	TuringSense	100	Robust unscented Kalman filter	S1: hand S2: forearm S3: upper arm S4: trunk	N–pose	Vicon	Flex./Ext. Abd./Add. IR.ER	Flex./Ext. Carrying angle Prona./Supina.	Flex.Ext. Abd./Add. IR/ER	Sun salutation
Wells et al.	2019	9	Xsens	100	Kalman filter	S1: upper arm S2: forearm	Anatomical calibration + functional calibration	Vicon	N/A	Flex,/Ext. Prona./Supina.	N/A	N/A
Wirth et al.	2019	10	DyCare Lynx	102.4	N/A	S1: hand S2: forearm	N/A	Vicon	N/A	N/A	Flex./Ext. Radial–ulnar dev.	N/A
Wu et al.	2022	7	Perception Neuron	100	N/A	S1: shoulders (upper portion of the scapula) S2: upper spine S3: upper arms (above the lateral elbow) S4: forearms (just above the lateral side of the wrist)	Static calibration	OptiTrack	Flex./Ext. Abd./Add. IR/ER	Flex./Ext. Prona./Supina.	N/A	Lifting box over the shoulder

Abbreviations: Abd., abduction; Add., adduction; ER, external rotation; Ext., extension; Flex., flexion; IR, internal rotation; Pron., pronation; Supi., supination; N/A, not applicable.

### 3.2. Risk of bias of the included studies

Seven articles were rated as HQ, 14 as MQ, 23 as LQ, and 7 as VLQ (Table 3). Agreement between both assessors was acceptable (Cohen’s kappa = 0.65; 95% CI = 0.59–0.71). More than half of the included articles received the highest scores in Q1 (Background & Research Question), Q8 (Protocol), and Q12 (Conclusion/Recommendations), but only three articles (5.7%) reported the complete sample size calculation process (Q5: Sample).

**Table 3.** Risk of bias assessment for included studies according to the Critical Appraisal of Study Design for Psychometric Articles

Study information	Study question	Study design					Measurement		Analyses			Rec.		Total		Quality
Author	Year	Q1	Q2	Q3	Q4	Q5	Q6	Q7	Q8	Q9	Q10	Q11	Q12	/24	%	
Abbasi–Kesbi et al.	2018	2	0	0	1	0	N/A	1	1	2	2	1	1	11	50.0	LQ
Alarcon–Aldana et al.	2022	2	0	1	1	0	N/A	2	2	2	1	0	2	13	59.1	LQ
Barreto et al.	2021	2	2	2	2	2	N/A	2	2	2	1	1	2	20	90.9	HQ
Bessone et al.	2022	2	1	1	1	1	N/A	2	2	1	2	2	2	17	77.3	MQ
Boddy et al.	2019	1	1	1	1	1	N/A	1	2	1	2	1	1	13	59.1	LQ
Callejas–Cuervo et al.	2017	1	0	0	1	0	N/A	1	1	0	1	0	1	6	27.3	VLQ
Camp et al.	2021	2	2	1	1	1	N/A	2	2	1	1	1	2	16	72.7	MQ
Chan et al.	2022	2	1	2	2	2	N/A	2	2	2	2	2	2	21	95.5	HQ
Chapman et al.	2019	2	1	2	1	1	N/A	1	2	2	1	1	1	13	59.1	LQ
Chen et al.	2020	1	2	1	0	1	N/A	2	2	1	0	0	1	11	50.0	LQ
Choo et al.	2022	2	2	2	2	1	N/A	1	2	2	2	2	1	19	86.4	HQ
Contreras et al.	2020	2	1	1	0	1	N/A	1	2	1	1	1	1	12	54.5	LQ
Digo et al.	2022	1	1	2	1	1	N/A	1	1	1	1	0	1	11	50.0	LQ
Dufour et al.	2021	1	1	1	1	1	N/A	2	2	1	1	1	1	13	59.1	LQ
Rovini et al.	2019	0	1	1	2	1	N/A	1	1	1	1	1	2	12	54.5	LQ
Ertzgaard et al.	2016	2	2	1	1	1	2	1	1	1	2	1	2	17	70.8	MQ
Esfahani et al.	2018	2	1	0	0	0	N/A	2	2	2	2	1	1	13	59.1	LQ
Fantozzi et al.	2016	2	2	1	2	1	N/A	2	2	1	1	1	2	17	77.2	MQ
Fischer et al.	2021	2	2	1	1	1	2	1	2	1	1	1	2	17	70.8	MQ
Goreham et al.	2022	1	1	1	2	1	N/A	2	2	2	1	2	1	16	72.7	MQ
Guignard et al.	2021	2	1	1	2	1	2	2	2	2	2	2	2	21	87.5	HQ
Henschke et al.	2022	2	2	2	2	1	N/A	2	2	2	2	1	2	20	90.9	HQ
Hubaut et al.	2022	1	2	1	0	1	N/A	2	2	1	1	1	1	13	59.1	LQ
Humadi et al.	2021	2	2	1	1	1	1	1	2	2	1	1	2	17	70.8	MQ
Laidig et al.	2017	0	0	0	0	0	N/A	1	0	1	0	0	1	3	13.6	VLQ
Ligorio et al.	2017	1	1	1	0	1	2	2	2	1	1	0	1	13	54.2	LQ
Ligorio et al.	2020	0	1	0	0	1	N/A	1	1	1	1	0	1	7	31.8	VLQ
Lin et al.	2021	2	2	0	1	1	2	2	2	1	1	1	2	17	70.8	MQ
Marta et al.	2020	1	0	1	1	0	N/A	1	1	2	0	0	1	8	36.4	VLQ
Mavor et al.	2020	2	1	0	1	1	N/A	1	1	1	0	1	2	11	50.0	LQ
Mihcin,et al.	2019	1	0	0	0	0	N/A	1	1	1	1	1	1	7	31.8	VLQ
Morrow et al.	2017	1	1	1	1	1	N/A	2	1	1	2	1	1	13	59.1	LQ
Muller et al.	2017	1	0	0	0	0	N/A	1	0	0	0	0	1	3	13.6	VLQ
Ohberg et al.	2019	2	2	1	1	1	2	1	1	1	2	1	2	17	70.8	MQ
Pedro et al.	2021	2	2	1	1	1	N/A	1	1	1	1	1	1	13	59.1	LQ
Picerno et al.	2019	1	1	1	1	1	N/A	1	1	2	1	1	2	13	59.1	LQ
Poitras et al.	2019	2	2	2	2	2	N/A	1	2	2	2	1	2	20	90.9	HQ
Robert–Lachaine et al.	2020	2	1	0	1	0	N/A	2	1	1	1	0	2	11	50.0	LQ
Robert–Lachaine et al.	2017b	2	2	0	1	1	N/A	1	2	2	2	1	2	16	72.7	MQ
Robert–Lachaine et al.	2017a	1	1	0	0	1	2	1	1	1	2	1	2	13	54.2	LQ
Ruiz–Malagon et al.	2022	2	2	1	1	1	2	2	2	2	2	1	2	20	83.3	MQ
Schall et al.	2016	1	1	1	1	1	2	2	2	2	1	1	2	17	70.8	MQ
Sers et al.	2020	1	2	1	1	1	N/A	2	2	2	2	1	2	17	77.3	MQ
Shepherd et al.	2017	1	1	1	1	1	N/A	1	1	1	1	1	1	11	50.0	LQ
Slade et al.	2022	2	2	1	1	1	N/A	2	2	1	1	1	2	16	72.7	MQ
Truppa et al.	2021a	1	1	0	0	1	N/A	1	0	1	0	0	1	6	27.3	VLQ
Truppa et al.	2021b	2	2	0	1	1	N/A	2	1	1	1	0	2	13	59.1	LQ
Truppa et al.	2022	2	1	1	0	1	N/A	2	1	2	0	0	1	11	50.0	LQ
Wells et al.	2019	1	1	0	0	1	N/A	2	2	1	1	0	1	11	50.0	LQ
Wirth et al.	2019	2	2	0	0	1	N/A	2	1	1	1	0	2	12	54.5	LQ
Wu et al.	2022	2	2	2	2	1	2	1	2	2	2	2	2	22	91.7	HQ

HQ, high quality; LQ, low quality; MQ, moderate quality; N/A, not applicable; VLQ, very low quality.

### 3.3. Characteristics of the wearable sensor

The common commercial IMU systems used were Xsens (*n* = 13), APDM/Opal (*n* = 4). and Perception Neuron (*n* = 4); it is worth noting that seven studies used customized IMU systems. A total of 32 papers reported the calibration process before data collection. Static anatomical calibration was performed often (*n* = 24), with dynamic anatomical calibration performed (*n* = 5).

### 3.4. Validity of the wearable sensors

Validity was assessed using Vicon system (*n* = 22), OptiTrack (*n* = 12), Qualisys (*n* = 6), Smart DX (*n* = 4), and other systems (*n* = 7) as reference systems. Although many statistical parameters related to the validity of IMUs were included in data extraction, we found through analysis that, due to the limitation of the number of studies and the inconsistency of the results reported, such as RMSE and LoA, there are only three statistics of mean ± SD, ICC, and Pearson’s *r* could be included in the meta-analysis. For studies not included in the meta-analysis, we also quantitatively summarized the extracted data in Supplementary file 3, and these data were used as supplements and references when discussing the results of the meta-analysis.

#### 3.4.1. Shoulder flexion/extension

**Figure 2.**
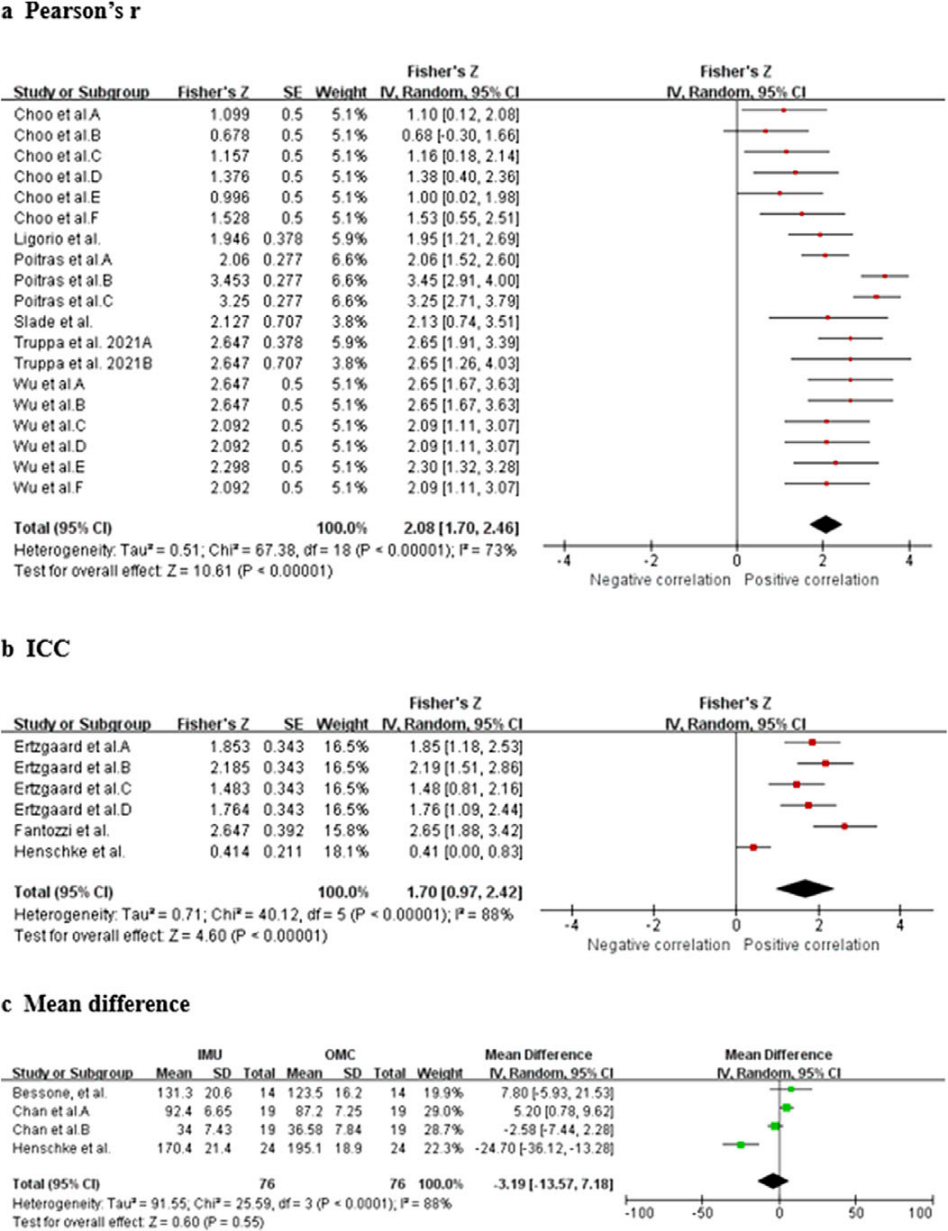
Shoulder flexion/extension. Forest plots showing the validity of shoulder flexion/extension measured using IMU. Red squares represent Fisher’s *Z*; green squares represent mean difference; bars indicate 95% CI and black diamonds as total data. Panel (a) describing the results of Pearson’s *r*: Choo et al. A (stationary walk), B (distance walk), C (stationary jog), D (distance jog), E (stationary wrist shot), F (distance wrist shot); Poitras et al. A (60° RoM), B (90° RoM), C (120° RoM); Wu et al. A (fast sample task (flexion)), B (slow simple task (flexion)), C (fast simple task (extension)), D (slow simple task (extension)), E (fast complex task), F (slow complex task). Panel (b) describing the results of ICC: Ertzgaard et al. A (cone task), B (throw task), C (coordination task one), D (coordination task two). Panel (c) describing the results of mean difference: Chan et al. A (flexion), B (extension). CI, confidence interval; IV, inverse variance; SE, standard error.

Data from seven studies (three HQ, two MQ to LQ, and two VLQ; Poitras et al., 2019; Ligorio et al., 2020; Truppa et al., 2021a,b; Choo et al., 2022; Slade et al., 2022; Wu et al., 2022) suggest that very strong relationship between IMU and OMC for shoulder flexion/extension measurements (total *n* = 60; *r* = 0.969, 95% CI [0.935, 0.986]; Tau^2^ = 0.51; *I* ^2^ = 73%) (Figure 2a). Sensitivity analysis showed that the results were robust even after excluding the study of Poitras et al. (2019) (*I* ^2^ = 43% and *r* = 0.954 [0.914, 0.976]). Based on the quality of the included studies, the level of evidence for this result is strong.

Data from three studies (one HQ and two MQ; Ertzgaard et al., 2016; Fantozzi et al., 2016; Henschke et al., 2022) suggested that excellent consistency between IMU and OMC for shoulder flexion/extension measurements (total *n* = 42; ICC = 0.935, 95% CI [0.749, 0.984]; Tau^2^ = 0.71; *I* ^2^ = 88%) (Figure 2b). Sensitivity analysis showed that, after excluding the study of Henschke et al. (2022), the *I* ^2^ decreased to 32% and ICC was changed to 0.961 [0.920, 0.982]. Sensitivity analysis showed that the results were robust. Based on the quality of the included studies, the level of evidence for this result is moderate.

Data from three studies (two HQ and one MQ; Bessone et al., 2022; Chan et al., 2022; Henschke et al., 2022) suggest that no significant measurement difference between IMU and OMC for shoulder flexion/extension measurements (total *n* = 57; mean difference = −3.19, 95% CI [−13.57, 7.18]; Tau^2^ = 91.55; *I* ^2^ = 88%; *Z* = 0.60 (*p* = 0.55)) (Figure 2c). A sensitivity analysis was conducted, which revealed that when the study of Henschke et al. (2022) was excluded, the *I* ^2^ reduced to 67% and the mean difference was 2.39 [−4.04, 8.82], with a *Z*-score of 0.73 (*p* = 0.47). This analysis demonstrated that the results were robust. Based on the quality of the included studies, the level of evidence for this result is strong.

#### 3.4.2. Shoulder abduction/adduction

**Figure 3.**
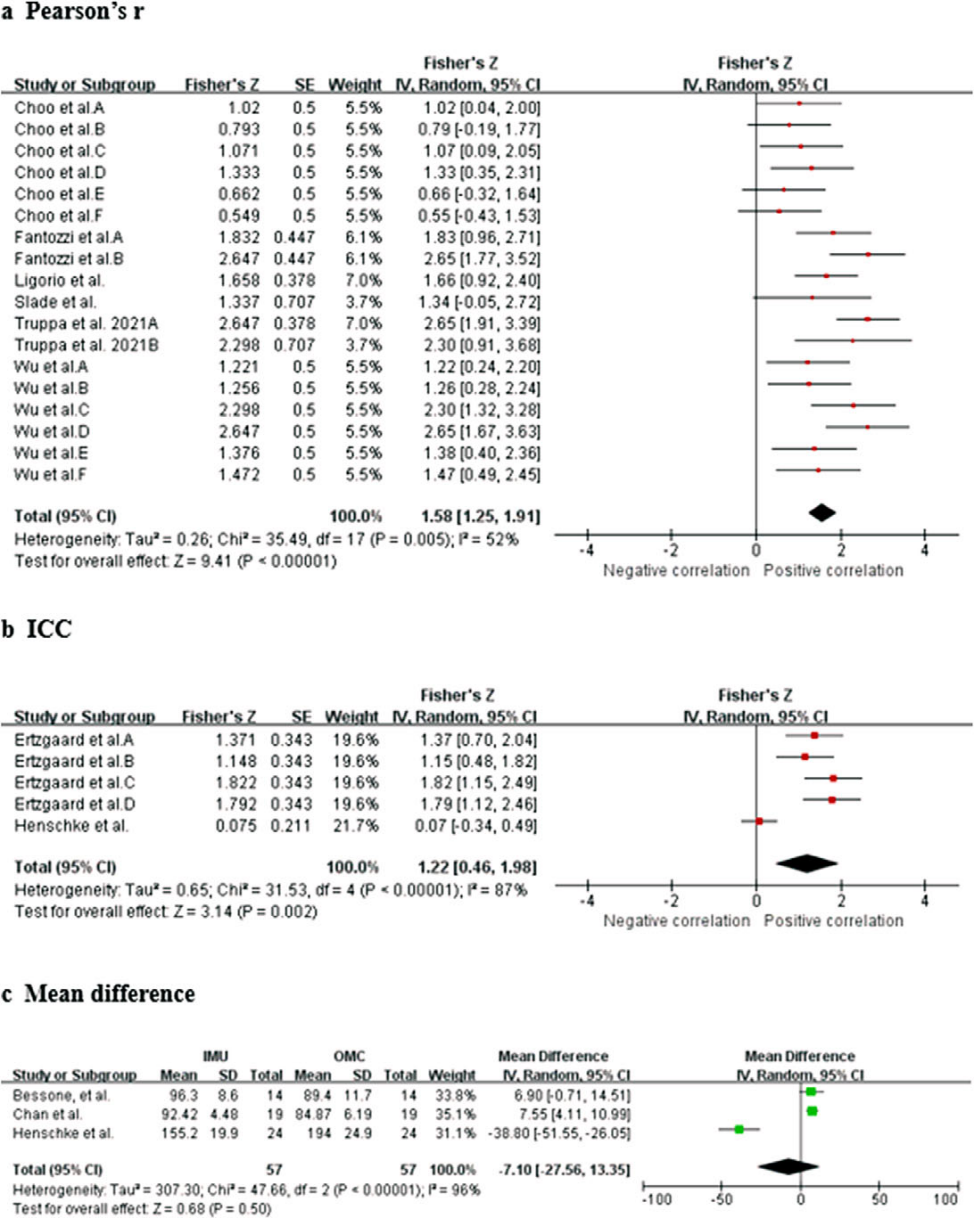
Shoulder abduction/adduction. Forest plots showing the validity of shoulder abduction/adduction measured using IMU. Red squares represent Fisher’s *Z*; green squares represent mean difference; bars indicate 95% CI and black diamonds as total data. Panel (a) describing the results of Pearson’s *r*: Choo et al. A (stationary walk), B (distance walk), C (stationary jog), D (distance jog), E (stationary wrist shot), F (distance wrist shot); Fantozzi et al. A (front-crawl task), B (breaststroke task); Wu et al. A (fast sample task (flexion)), B (slow simple task (flexion)), C (fast simple task (extension)), D (slow simple task (extension)), E (fast complex task), F (slow complex task). Panel (b) describing the results of ICC: Ertzgaard et al. A (cone task), B (throw task), C (coordination task one), D (coordination task two). Panel (c) describing the results of mean difference. CI, confidence interval; IV, inverse variance; SD, standard deviation; SE, standard error.

Data from seven studies (two HQ, three MQ to LQ, and two VLQ; Fantozzi et al., 2016; Ligorio et al., 2020; Truppa et al., 2021a,b; Choo et al., 2022; Slade et al., 2022; Wu et al., 2022) suggest that very strong relationship between IMU and OMC for shoulder abduction/adduction measurements (total *n* = 52; *r* = 0.919, 95% CI [0.848, 0.957]; Tau^2^ = 0.29; *I* ^2^ = 52%) (Figure 3a). Upon conducting a sensitivity analysis and excluding the study of Truppa et al. (2021a), the *I* ^2^ decreased to 41%, while the Pearson’s *r* remained high at 0.905 with a confidence interval of [0.831, 0.948]. Sensitivity analysis showed that the results were robust. Based on the quality of the included studies, the level of evidence for this result is strong.

Data from two studies (one HQ and one MQ; Ertzgaard et al., 2016; Henschke et al., 2022) suggest that good consistency between IMU and OMC for shoulder abduction/adduction measurements (total *n* = 34; ICC = 0.840, 95% CI [0.430, 0.963]; Tau^2^ = 0.65; *I*^2^ = 87%) (Figure 3b). Sensitivity analyses could not be performed due to the insufficient number of studies. Based on the quality of the included studies, the level of evidence for this result is moderate.

Data from three studies (two HQ and one MQ; Bessone et al., 2022; Chan et al., 2022; Henschke et al., 2022) suggest that no significant measurement difference between IMU and OMC for shoulder abduction/adduction measurements (total *n* = 57; mean difference = −7.10, 95% CI [−27.56, 13.35]; Tau^2^ = 307.30; *I* ^2^ = 96%; *Z* = 0.68 (*p* = 0.50)) (Figure 3c). After excluding the study of Henschke et al. (2022), sensitivity analysis revealed that the *I* ^2^ reduced to 0% and the mean difference was 7.44 [4.31, 10.57], with *Z*-score of 4.66 (*p* < 0.00001). Sensitivity analysis showed that the results were not robust. Based on the quality of the included studies, the level of evidence for this result is strong.

#### 3.4.3. Shoulder internal/external rotation

**Figure 4.**
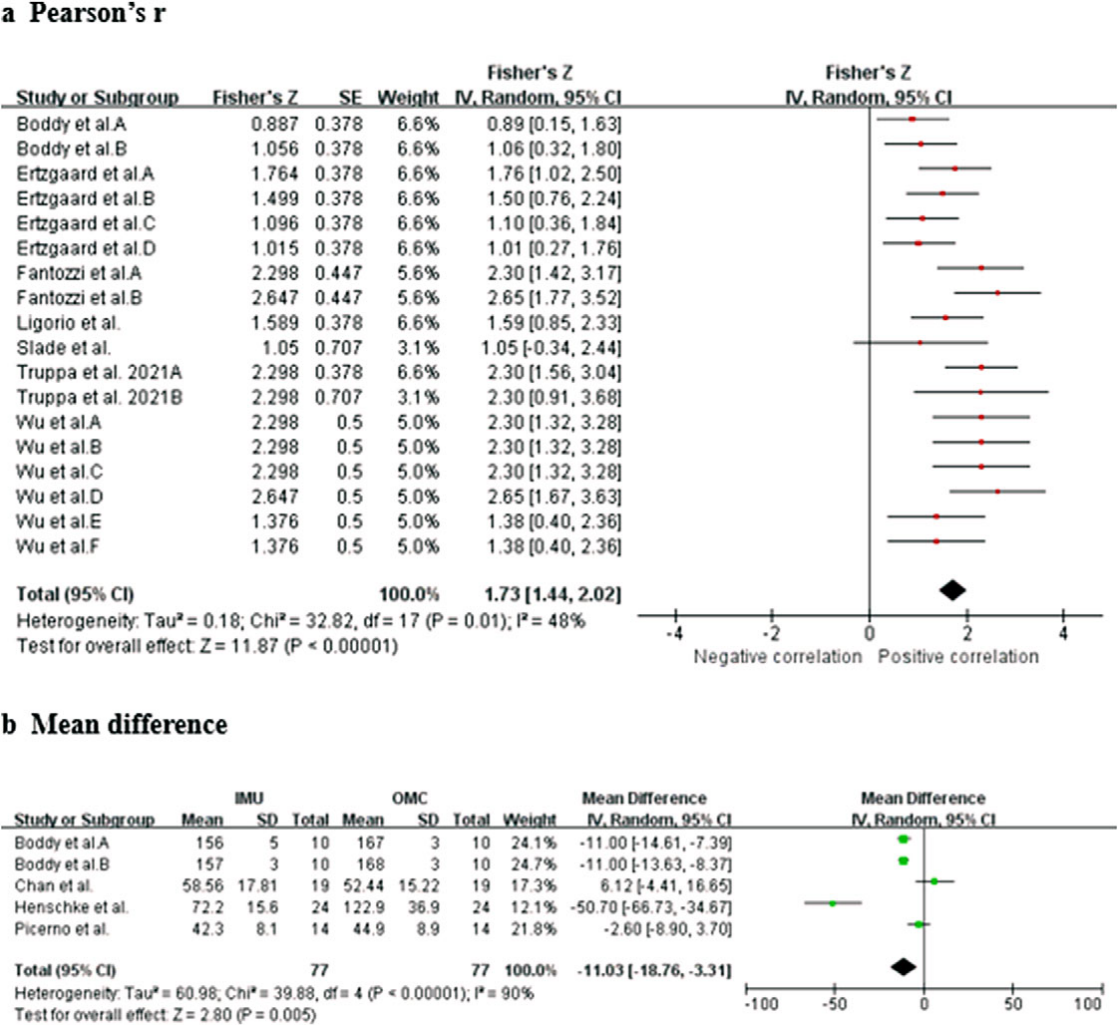
Shoulder internal/external rotation. Forest plots showing the validity of shoulder rotation measured using IMU. Red squares represent Fisher’s *Z*; green squares represent mean difference; bars indicate 95% CI and black diamonds as total data. Panel (a) describing the results of Pearson’s *r*: Boddy et al. (2019) A (fastball), B (off-speed); Ertzgaard et al. A (cone task), B (throw task), C (coordination task one), D (coordination task two); Fantozzi et al. A (front-crawl task), B (breaststroke task); Wu et al. A (fast sample task (flexion)), B (slow simple task (flexion)), C (fast simple task (extension)), D (slow simple task (extension)), E (fast complex task), F (slow complex task). Panel (b) describing the results of mean difference: Boddy et al. A (fastball), B (off-speed). CI, confidence interval; IV, inverse variance; SD, standard deviation; SE, standard error.

Data from seven studies (one HQ, four MQ to LQ, and two VLQ; Ertzgaard et al., 2016; Fantozzi et al., 2016; Boddy et al., 2019; Ligorio et al., 2020; Truppa et al., 2021a,b; Slade et al., 2022; Wu et al., 2022) suggest that very strong relationship between IMU and OMC for shoulder internal/external rotation measurements (total *n* = 64; *r* = 0.939, 95% CI [0.894, 0.965]; Tau^2^ = 0.18; *I* ^2^ = 48%) (Figure 4a). Based on the quality of the included studies, the level of evidence for this result is moderate.

Data from four studies (two HQ and two LQ; Boddy et al., 2019; Picerno et al., 2019; Chan et al., 2022; Henschke et al., 2022) suggest that significant measurement difference between IMU and OMC for shoulder internal/external rotation measurements (total *n* = 67; mean difference = −11.03, 95% CI [−18.76, −3.31]; Tau^2^ = 60.98; *I* ^2^ = 90%; *Z* = 2.80 (*p* = 0.005)) (Figure 4b). Sensitivity analysis showed that after excluding the study of Chan et al. (2022) and Henschke et al. (2022), the *I* ^2^ value decreased to 67% and the mean difference was −9.13 [−13.09, −5.17], with *Z*-score of 4.52 (*p* < 0.00001). Sensitivity analysis showed that the results were robust. Based on the quality of the included studies, the level of evidence for this result is strong.

#### 3.4.4. Elbow flexion/extension

**Figure 5.**
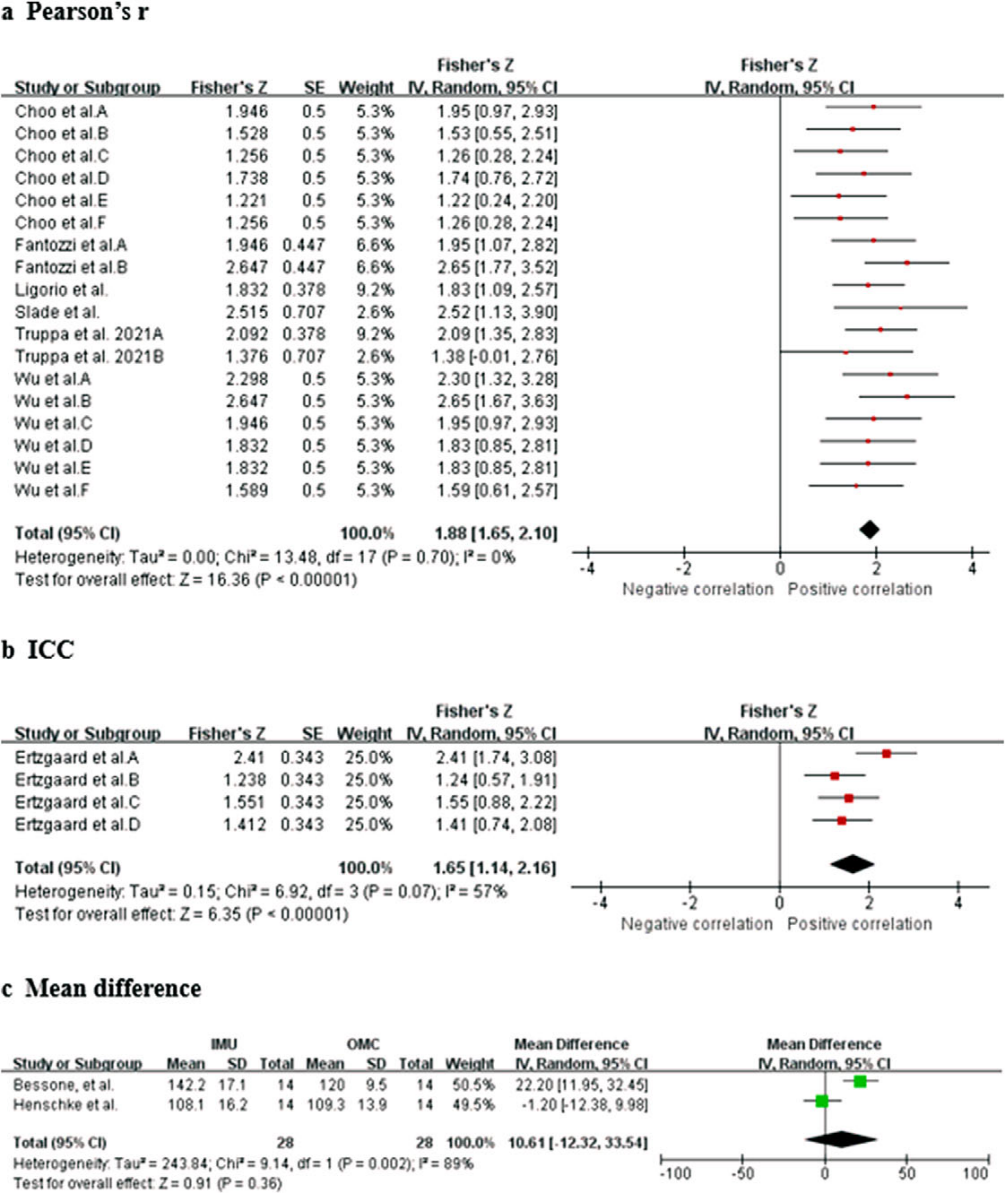
Elbow flexion/extension. Forest plots showing the validity of elbow flexion/extension measured using IMU. Red squares represent Fisher’s *Z*; green squares represent mean difference; bars indicate 95% CI and black diamonds as total data. Panel (a) describing the results of Pearson’s *r*: Choo et al. A (stationary walk), B (distance walk), C (stationary jog), D (distance jog), E (stationary wrist shot), F (distance wrist shot); Fantozzi et al. A (front-crawl task), B (breaststroke task); Wu et al. A (fast sample task (flexion)), B (slow simple task (flexion)), C (fast simple task (extension)), D (slow simple task (extension)), E (fast complex task), F (slow complex task). Panel (b) describing the results of ICC: Ertzgaard et al. A (cone task), B (throw task), C (coordination task one), D (coordination task two). Panel (c) describing the results of mean difference. CI, confidence interval; IV, inverse variance; SD, standard deviation; SE, standard error.

Data from seven studies (two HQ, three MQ to LQ, and two VLQ; Fantozzi et al., 2016; Ligorio et al., 2020; Truppa et al., 2021a,b; Choo et al., 2022; Slade et al., 2022; Wu et al., 2022) suggest that very strong relationship between IMU and OMC for elbow flexion/extension measurements (total *n* = 52; *r* = 0.954, 95% CI [0.929, 0.970]; Tau^2^ = 0.00; *I* ^2^ = 0%) (Figure 5a). Based on the quality of the included studies, the level of evidence for this result is strong.

Data from one MQ study (Ertzgaard et al., 2016) suggest that excellent consistency between IMU and OMC for elbow flex./ext. measurements (total *n* = 10; ICC = 0.929, 95% CI [0.814, 0.974]; Tau^2^ = 0.15; *I* ^2^ = 57%). Based on the quality of the included studies, the level of evidence for this result is limited. Data from two studies (one HQ and one MQ; Bessone et al., 2022; Henschke et al., 2022) suggest that no significant measurement difference between IMU and OMC for shoulder flexion/extension measurements (total *n* = 38; mean difference = 10.61, 95% CI [−12.32, 33.54]; Tau^2^ = 243.84; *I* ^2^ = 89%; *Z* = 0.91 (*p* = 0.36)) (Figure 5b). Sensitivity analyses could not be performed due to the insufficient number of studies. Based on the quality of the included studies, the level of evidence for this result is moderate.

#### 3.4.5. Elbow pronation/supination

**Figure 6.**
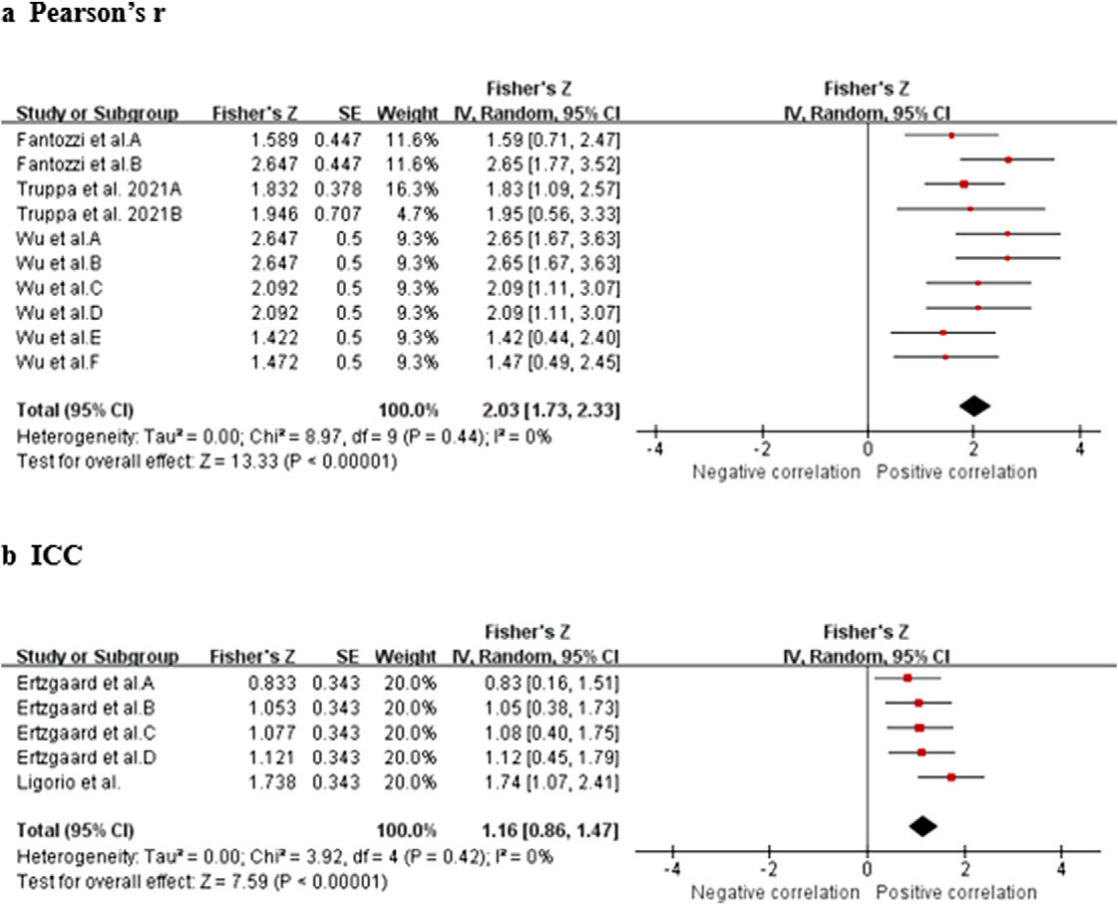
Elbow pronation/supination. Forest plots showing the validity of elbow pronation/supination measured using IMU. Red squares represent Fisher’s *Z*; green squares represent mean difference; bars indicate 95% CI and black diamonds as total data. Panel (a) describing the results of Pearson’s *r*: Fantozzi et al. A (front-crawl task), B (breaststroke task); Wu et al. A (fast sample task (flexion)), B (slow simple task (flexion)), C (fast simple task (extension)), D (slow simple task (extension)), E (fast complex task), F (slow complex task). Panel (b) describing the results of ICC: Ertzgaard et al. A (cone task), B (throw task), C (coordination task one), D (coordination task two). CI, confidence interval; IV, inverse variance; SE, standard error.

Data from four studies (one HQ, two MQ to LQ, and one VLQ; Fantozzi et al., 2016; Truppa et al., 2021a,b; Wu et al., 2022) suggest that very strong relationship between IMU and OMC for elbow pronation/supination measurements (total *n* = 30; *r* = 0.966, 95% CI [0.939, 0.981]; Tau^2^ = 0.00; *I* ^2^ = 0%) (Figure 6a). Based on the quality of the included studies, the level of evidence for this result is moderate. Data from two studies (Ertzgaard et al., 2016; Ligorio et al., 2020) suggest that good consistency between IMU and OMC for elbow pronation/supination measurements (total *n* = 20; ICC = 0.821, 95% CI [0.696, 0.900]; Tau^2^ = 0.00; *I* ^2^ = 0%) (Figure 6b). Based on the quality of the included studies, the level of evidence for this result is limited.

#### 3.4.6. Wrist flexion/extension

**Figure 7.**
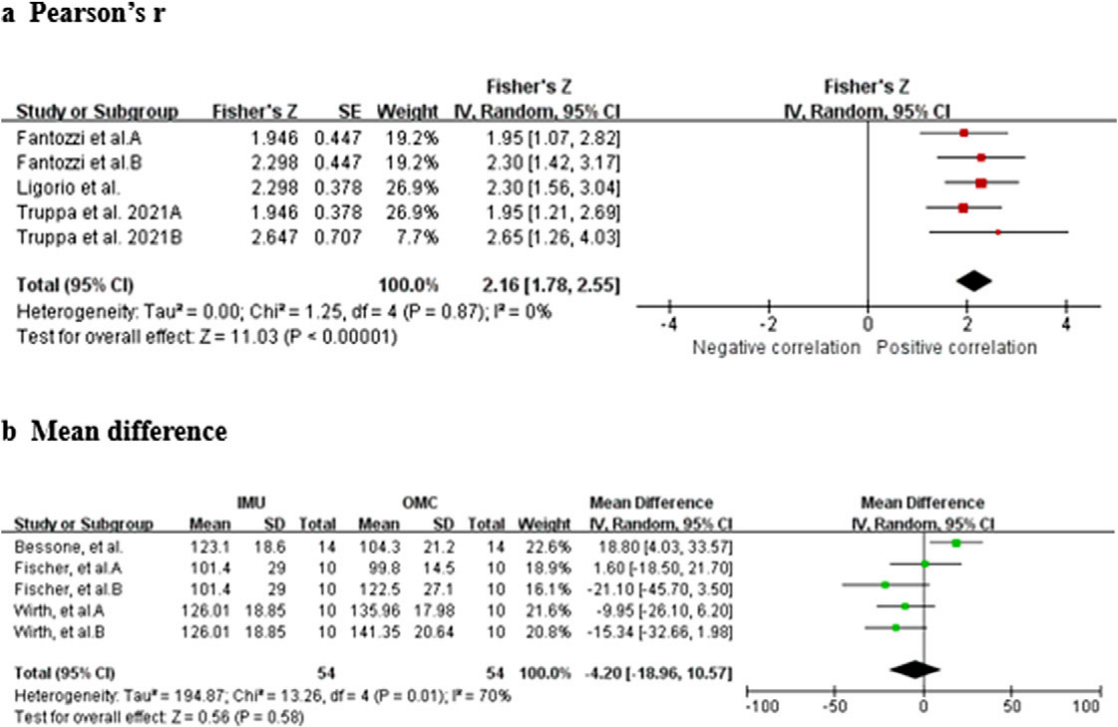
Wrist flexion/extension. Forest plots showing the validity of wrist flexion/extension measured using IMU. Red squares represent Fisher’s *Z*; green squares represent mean difference; bars indicate 95% CI and black diamonds as total data. Panel (a) describing the results of Pearson’s *r*: Fantozzi et al. A (front-crawl task), B (breaststroke task). Panel (b) describing the results of mean difference: Wirth et al. A (marker on the skin), B (marker on the sensor); Fischer et al. A (marker on the skin), B (marker on the sensor). CI, confidence interval; IV, inverse variance; SD, standard deviation; SE, standard error.

Data from four studies (two MQ to LQ and two VLQ; Fantozzi et al., 2016; Ligorio et al., 2020; Truppa et al., 2021a,b) suggest that very strong relationship between IMU and OMC for wrist flex./ext. measurements (total *n* = 33; *r* = 0.974, 95% CI [0.945, 0.988]; Tau^2^ = 0.00; *I* ^2^ = 0%) (Figure 7a). Based on the quality of the included studies, the level of evidence for this result is moderate. Data from three studies (two MQ and one LQ; Wirth et al., 2019; Fischer et al., 2021; Bessone et al., 2022) suggest that no significant measurement difference between IMU and OMC for wrist flex./ext. measurements (total *n* = 34; mean difference = −4.20, 95% CI [−18.96, 10.57]; Tau^2^ = 194.87; *I* ^2^ = 70%; *Z* = 0.56 (*p* = 0.58)) (Figure 7b). Sensitivity analysis showed that after excluding the study of Bessone et al. (2022), the *I* ^2^ value decreased to 0%. Furthermore, the mean difference was −10.64 with a 95% confidence interval of [−20.05, −1.23], *Z*-score of 2.22 (*p* = 0.03). Sensitivity analysis showed that the results were not robust. Based on the quality of the included studies, the level of evidence for this result is moderate.

#### 3.4.7. Wrist ulnar/radial deviation

**Figure 8.**
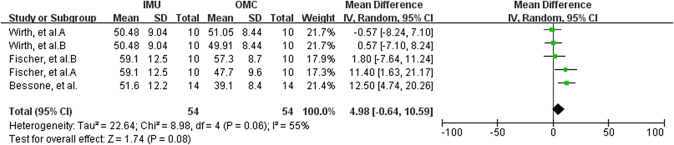
Wrist ulnar/radial deviation. Forest plot showing the validity of wrist ulnar/radial deviation measured using IMU. Green squares represent mean difference; bars indicate 95% CI and black diamonds as total data. Figure describing the results of mean difference: Wirth et al. A (marker on the skin), B (marker on the sensor); Fischer et al. A (marker on the skin), B (marker on the sensor). CI, confidence interval; IV, inverse variance; SD, standard deviation.

Data from three studies (two MQ and one LQ; Wirth et al., 2019; Fischer et al., 2021; Bessone et al., 2022) suggest that the validity for wrist ulnar/radial deviation measured with IMUs (total *n* = 34; mean difference = 4.98, 95% CI [−0.64, 10.59]; Tau^2^ = 22.64; *I* ^2^ = 55%; *Z* = 1.74 (*p* = 0.08)) (Figure 8). Sensitivity analysis showed that after excluding the study of Wirth et al. (2019), the *I* ^2^ reduced to 38%, and the mean difference was 8.85 [2.27, 15.42], with *Z*-score of 2.64 (*p* = 0.008). Sensitivity analysis showed that the results were not robust. Based on the quality of the included studies, the level of evidence for this result is moderate.

### 3.5. Subgroup analysis

The selection of subgroup analysis is mainly based on two key considerations. First, the subgroup classification needs to have practical significance and may affect the validity of IMU measurement, such as different fusion algorithms, the complexity of measured motion, and so forth. Second, there needs to be sufficient sample size for the corresponding subgroup (at least three studies and no less than 20 subjects). By reading the included literature and comparing the characteristics of different studies, this study mainly conducts subgroup analysis based on two different classifications: complexity of task and placements of markers. However, it is important to note a limitation regarding the fusion algorithms, which are crucial for the data processing of IMUs. We observed that most studies did not provide detailed reports on the algorithm parameters used. This lack of detailed reporting hindered our ability to effectively generalize and categorize the algorithms for subgroup analysis. As a result, the fusion algorithms could not be classified as a separate subgroup in this study.

#### 3.5.1. Complexity of motion task

Based on the complexity of upper limb motor tasks, the included motor tasks in this study were categorized as either complex tasks (CTs) or simple tasks (STs). The CTs were defined as upper limb movements that involved multiple planes of motion, such as baseball pitching or moving objects. The STs referred to upper limb movements that occurred in only one plane of motion, such as simple flexion and extension of the shoulder joint. Furthermore, the arm swing motion of the upper limb during walking, which is periodic and mostly occurs in the sagittal plane, was also classified as a simple motion task.

**Figure 9.**
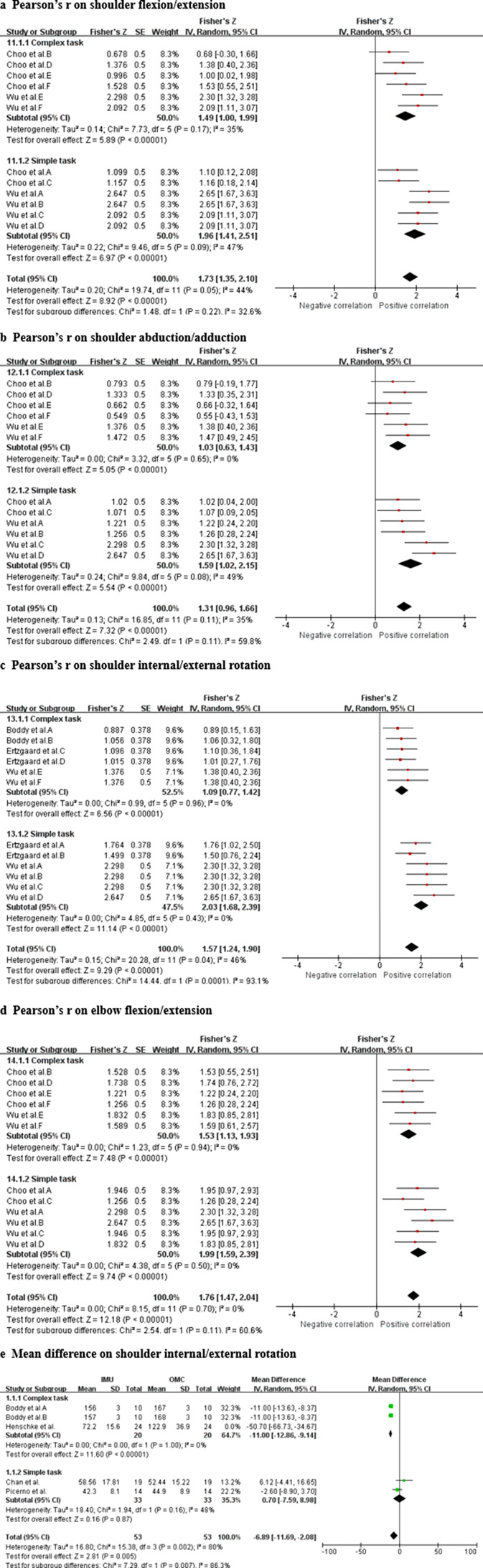
Complexity of motion task. Subgroup analysis showing the validity of the IMU for measuring joint range of motion at different motion task complexities. Red squares represent Fisher’s *Z*; green squares represent mean difference; bars indicate 95% CI and black diamonds as total data. Panel (a) describing the results of Pearson’s *r* for measuring shoulder flexion/extension. Panel (b) describing the results of Pearson’s *r* for measuring shoulder abduction/adduction. Panel (c) describing the results of Pearson’s *r* for measuring shoulder internal/external rotation. Panel (d) describing the results of Pearson’s *r* for measuring elbow flexion/extension. Panel (e) describing the results of mean difference for measuring shoulder internal/external rotation.

The results of subgroup analysis showed that the validity of IMU in measuring shoulder flexion/extension under complex motor tasks is the same as that of simple motor tasks (CT: Pearson’s *r* = 0.903 [0.762, 0.963], ST: Pearson’s *r* = 0.961 [0.887, 0.987]) (Figure 9a). The IMU has less validity in measuring shoulder abduction/adduction in complex motion tasks than simple motion tasks (CT: Pearson’s *r* = 0.774 [0.558, 0.892], ST: Pearson’s *r* = 0.920 [0.770, 0.973]) (Figure 9b). The IMU has less validity in measuring shoulder internal/external rotation in complex motion tasks than simple motion tasks (CT: Pearson’s *r* = 0.797 [0.647, 0.890], ST: Pearson’s *r* = 0.966 [0.933, 0.983]) (Figure 9c). The validity of IMU in measuring elbow flexion/extension under complex motor tasks is the same as that of simple motor tasks (CT: Pearson’s *r* = 0.910 [0.811, 0.959], ST: Pearson’s *r* = 0.963 [0.920, 0.983]) (Figure 9d). The results of subgroup analysis showed that for shoulder internal/external rotation, both CTs and STs shown significant mean difference between IMU and OMC measurements (*p* < 0.00001 and 0.005, respectively) (Figure 9e).

#### 3.5.2. Placements of markers

**Figure 10.**
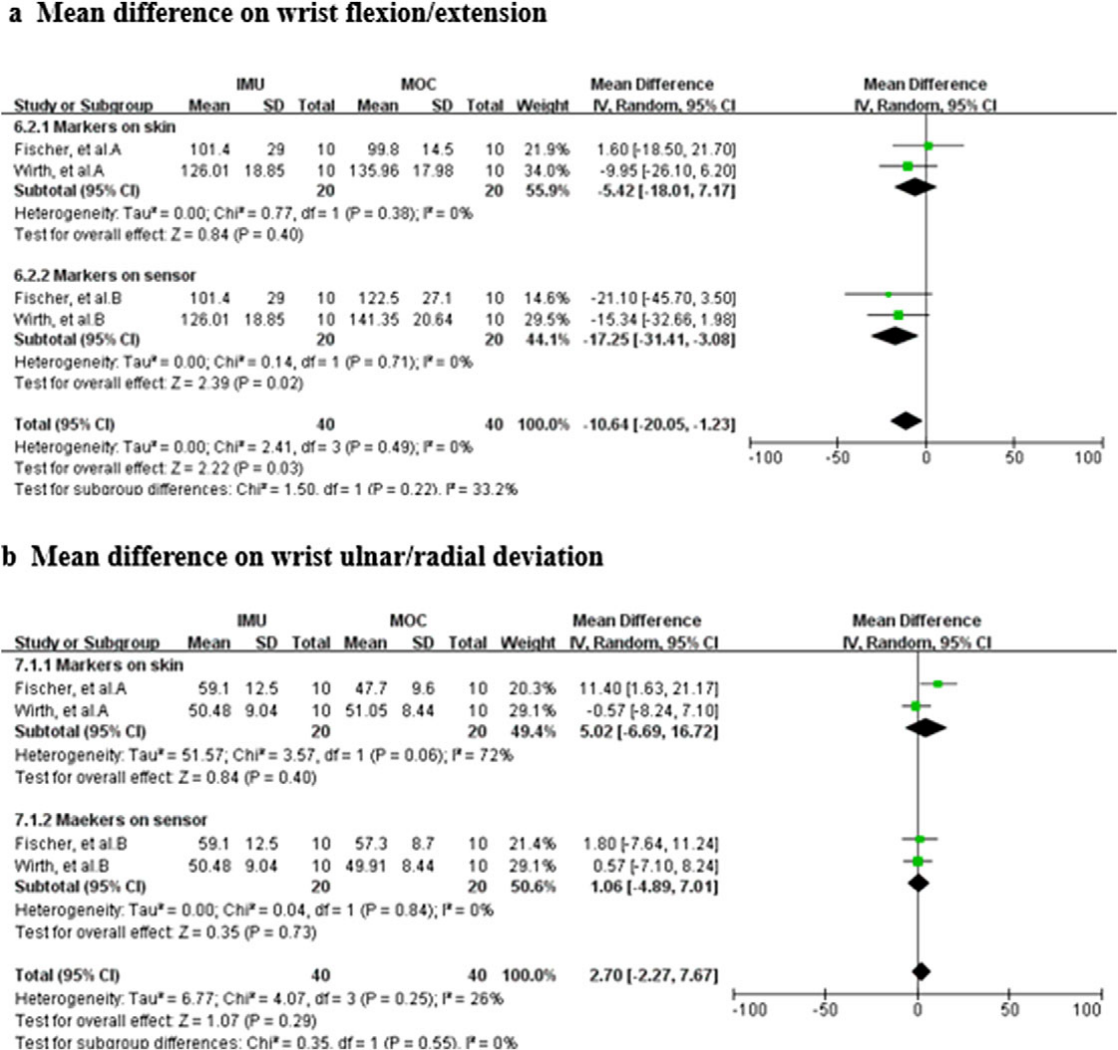
Placement of markers. Subgroup analysis showing the validity of the IMU for measuring joint range of motion at different placement of markers. Green squares represent mean difference; bars indicate 95% CI and black diamonds as total data. Panel (a) describing the results of mean difference for measuring wrist flexion/extension. Panel (b) describing the results of mean difference for measuring wrist ulnar/radial deviation.

The vast majority of included studies used standard marker placement, but two studies (Wirth et al., 2019; Fischer et al., 2021) compared joint RoM measurements when markers were placed on the skin and on the sensors. The results of subgroup analysis showed that for wrist flexion/extension, when the marker was placed on the sensor, there was a statistically significant difference in the joint RoM measurements between the IMU and the OMC (mean difference = −17.25 [−31.41, −3.08], *Z* = 2.39 (*p* = 0.02)), whereas when the marker was placed on the skin, there was no statistically significant difference between the two measurements (mean difference = −5.42 [−18.01, 7.17], *Z* = 0.84 (*p* = 0.40)) (Figure 10a). For wrist ulnar/radial deviation, neither marker on skin nor on sensors shown significant difference (*p* = 0.40 and 0.73, respectively) (Figure 10b).

## 4. Discussions

### 4.1. Principal findings

This systematic review provided an overview of the characteristics of IMUs used to measure upper extremity motion, and evaluates their concurrent validity compared to marker-based motion capture systems in measuring RoM of upper extremity joints. A total of 51 articles were included in this review, and the data in the literature were quantitatively integrated and meta-analyzed. To the best of our knowledge, this is the first meta-analysis study on the validity of IMU measurements of upper extremity RoM, and as such, there is a scarcity of relevant references pertaining to methodology and data extraction. The research methodology employed in this article was primarily based on the meta-analysis process recommended by PRISMA, as well as previous systematic review and/or meta-analysis studies (Walmsley et al., 2018; Kobsar et al., 2020; Zeng et al., 2022) focused on IMUs measuring kinematic parameters of the lower and upper extremities.

Unlike marker-based motion capture systems, there is no consensus on where to place IMUs when measuring human kinematic parameters. The previous studies had the same findings. The systematic reviews of Kobsar et al. (2020) and Zeng et al. (2022) both pointed out that, when measuring the kinematic parameters of the lower limbs, the IMU placements reported in the relevant literature were varied, and similar conclusions also appeared in the kinematic measurements of the upper limbs. In this review, the placement of IMUs was described differently across studies, even for the same brand of IMU. Indeed, we found that some commercial IMUs only provide limited or vague descriptions of anatomical positions, such as upper arm or forearm, to guide placements. Without a relatively uniform IMU placements specification, measurement inconsistency will inevitably be introduced.

Calibration methods for IMU systems are also inconsistent. Most studies use static calibration, also called anatomical calibration, as recommended by manufacturers, the main purpose of which is to establish an anatomical reference system for joints, such as T-pose (participants to have shoulders abducted by 90° with the palms facing the floor) and S-pose (participants to bend knees approximately 45° and place arms in front and position them parallel to the floor). Dynamic calibration, also known as functional calibration, is a customized calibration method based on the joint motion pattern to be measured and can be used to estimate the joint rotation axis; for example, when measuring the movement of the elbow joint, it is necessary to calibrate the flexion–extension and pronation–supination axes in advance. Most of the literature claiming to use dynamic calibration did not describe the specific calibration method, making the methodological lack of reproducibility. In addition, a study (Ligorio et al., 2017) compared the impact of anatomical calibration and dynamic calibration on the accuracy of IMU measurement of elbow joint angles, and found that dynamic calibration is more targeted, which indicated that functional calibration methods are more accurate than anatomical methods when estimating the elbow joint angle.

Additionally, three main types of data fusion algorithms are used for IMU data processing: Kalman, complementary and customized algorithms. Classical Kalman algorithm is one of the most common models to reduce noise from sensor signals, and it is based on recursive Bayesian filtering, while the noise is assumed Gaussian (Marta et al., 2020). Some other filters based on the Kaman algorithms, such as extended Kalman algorithm (EKF; Truppa et al., 2021), are also used to process IMU signals. However, Kalman filter has a complex mathematical model, which is not friendly to non-professionals, so the simpler complementary filters appeared. Complementary filters include both low-pass and high-pass filters. The low-pass filter removes high-frequency noise like the accelerometer in the case of vibration, and high-pass filter removes low-frequency noise such as the drift of the gyroscope. Chen et al. (2020) compared the validity of four data fusion algorithms in the IMU measurement of upper limb kinematics, including Kalman and complementary filters. The results showed that compared with the reference system (OMC), the measurement errors of the peak joint angles of the four algorithms were all less than 4.5°, and the authors believed that the complementary filters were comparable to the more complex Kalman filters. However, data fusion algorithms were not reported in nearly half of the included literature (43%), and the reason may be that most commercial wearable sensors use built-in signal processing software with embedded algorithms, so the user does not know the type of algorithm used. In addition, many literatures used various custom algorithms (Truppa et al., 2021, 2022; Yang et al., 2022), and this inconsistence is similar to previous review article (Zeng et al., 2022) and makes it difficult to quantitatively compare the impact of different algorithms on the measurement validity of IMUs.

This review focuses on the validity of IMUs in measuring RoM in the three major joints and seven degrees of freedom (shoulder: 3DOF, elbow: 2DOF, and wrist: 2DOF) of the upper extremity in adults. Similar to previous reviews (Walmsley et al., 2018; Poitras et al., 2019), this review found that IMUs have high validity in measuring sagittal motion (flexion and extension) of the upper extremity joints. However, IMUs showed less validity in measuring shoulder adduction–abduction and elbow pronation–supination, albeit within an acceptable range. The level of evidence for the above results is moderate and/or strong. Notably, the results for the shoulder rotation were conflicting. Pearson’s *r* showed excellent agreement between IMU and OMC, but there was a statistically significant difference in the mean difference between the two measurement systems. In addition, although the meta-analysis result showed that there was no significant difference between the IMU and OMC systems in measuring the ulnar-radial deviation of the wrist joint, the *p-*value was 0.08, so we could not draw a firm conclusion, and more high-quality studies are needed in the future. As mentioned above, many included literatures only reported statistical parameters such as RMSE and LoA. Due to the lack of homogeneity of these results, meta-analysis could not be performed. In this review, the RMSE for 3DOF of shoulder were all of 16° or less (Callejas-Cuervo et al., 2017; Mavor et al., 2020; Lin et al., 2021; Bessone et al., 2022; Choo et al., 2022), the RMSE for flexion–extension of elbow was from 1.9° to 27.1° and for pronation–supination of elbow was from 6° to 16.7° (Fantozzi et al., 2016; Mavor et al., 2020; Bessone et al., 2022). These findings are basically consistent with those reported by Poitras et al. (2019).

Although this review did not discuss the reliability of IMUs for measuring upper extremity RoM, this has been reported in previous systematic reviews (Walmsley et al., 2018; Poitras et al., 2019). Walmsley et al. (2018) reported that adequate to excellent agreement for 2DOF at the shoulder (ICC 0.68–0.81), poor to moderate agreement for the 2DOF at the elbow (ICC 0.16–0.83), and the highest overall agreement with ICC values ranging from 0.65 to 0.89 for 2DOF at wrist. Similar conclusions can be found in the article by Poitras et al. (2019), the results show poor to good reliability (ICC = 0.2 to 0.77) at elbow and good to excellent intra-rater reliability for all joint movements (CMC and ICC between 0.79 and 0.96). However, compared with validity, there are fewer literatures on the reliability of IMU measurement results, and only a few references are included in the above-mentioned review articles, so the level of evidence for these conclusions is insufficient.

The complexity of motion may impact the validity of IMU measurements. Subgroup analysis revealed that the validity of IMU measurements in complex movements was lower than in simple movements, particularly in adduction–abduction and internal–external rotation of the shoulder joint, with moderate agreements between IMU and OMC measurements. This conclusion is supported by the findings of Walmsley et al. (2018). Future research is needed to explore factors that influence the validity of IMU measurements in complex movements, and to develop appropriate strategies for enhancing the accuracy of IMU-based measurements in these scenarios.

We employed sensitivity analysis to mitigate heterogeneity in our meta-analysis. The major source of heterogeneity stems from three specific literature sources (Wirth et al., 2019; Bessone et al., 2022; Henschke et al., 2022). Upon further examination of the full text, it was revealed that these three studies utilized IMUs from less well-known brands that lack sufficient reliability and validity testing on large samples. Consequently, the research conducted in these sources is considered exploratory psychometrics, and the findings can serve to enhance the performance of IMU products. Some wearable sensors are primarily designed for use by clinicians and physical therapists, and their measurement accuracy may not satisfy laboratory requirements. Therefore, it is advisable for laboratory users to assess whether there are any psychometric research reports available on the IMU system currently in use. In the sensitivity analysis focusing on the mean difference of shoulder flexion/extension and rotation, the value of *I* ^2^ decreased but did not fall below 50% after certain studies were excluded. This suggests persistent moderate heterogeneity among the remaining studies. Consequently, the findings in these two parts should be approached with caution, acknowledging the continued presence of variability across the studies.

The RoM calculation is predicated on the disparity between peak joint angles. However, a potential issue is that it is still possible to obtain the same RoM when the peak joint angles measured by the IMU differ greatly from the reference system. While few of the studies we evaluated provided peak joint angle data, some sources furnished absolute joint angle curves for both IMU and OMC systems, with most curves indicating that the measurement curves for the two systems were relatively comparable. Nevertheless, Bessone et al. (2022) conducted a comparison of two systems, Vicon (OMC) and aktos-t (IMU), and discovered that while moderate to good agreements were noted for measuring the total RoM of the shoulder and elbow joints, there was a significant disparity in the measurement of peak angle of motion for both joints. Although our systematic review’s findings offer supportive evidence for the validity of IMU-based measurement of upper extremity RoM, further research is required to ascertain the accuracy of IMU-based peak joint angle measurement.

In practical applications, the inherent limitations and drawbacks of IMUs cannot be overlooked. A notable issue with IMUs is drift, which is a gradual deviation in the sensor’s measurements over time, especially evident during the process of integrating acceleration data to calculate velocity and position, leading to cumulative errors. Additionally, IMUs are sensitive to environmental influences such as temperature changes, electromagnetic fields, and vibrations, all of which can significantly compromise sensor accuracy. Another challenge with IMUs, particularly in wearable applications, is their limited operational duration due to reliance on battery power. This constraint becomes a significant issue in long-term monitoring or tracking tasks. Moreover, the size and design of the IMU device could impact user comfort and acceptance, especially in scenarios requiring extended wearing. Finally, while IMUs are adept at measuring acceleration and rotational changes, they do not directly provide spatial information. Obtaining positional data requires additional processing and integration, potentially introducing further errors.

### 4.2. Recommendations for future studies

Finally, it is worth noting that over half of the studies we included were deemed to have low or very low methodological quality, a finding that aligns with the results of certain prior systematic reviews (Walmsley et al., 2018; Poitras et al., 2019; Kobsar et al., 2020; Zeng et al., 2022). Furthermore, most of the literature we reviewed did not furnish information on the sample size estimation method, and over half of the research samples comprised 10 participants or fewer, further compromising the robustness of the conclusions drawn from this study. Of equal importance, the lack of standardized reporting guidelines for validity outcomes has led to substantial differences in statistical parameters among the included literature, thereby limiting the number of sources that can be leveraged for data integration and meta-analysis. As such, future research must seek to enhance the methodological quality of their investigations by considering the aforementioned findings.

### 4.3. Review limitations

This systematic review solely examined the validity of IMU technology in the measurement of upper extremity joint RoM and did not address reliability. Additionally, since all the literature we reviewed comprised comparative studies of IMU and marker-based motion capture systems, the applicability of IMUs is confined to laboratory settings. Consequently, the conclusions of our study cannot be readily extrapolated to assess the measurement performance of IMUs in real-world working environments. Future reviews should instead aim to evaluate the measurement performance of IMUs in non-laboratory settings.

Given the rapid pace of updates and iterations in commercial IMU systems, coupled with the existence of a prior systematic review summarizing relevant research prior to 2016 (Walmsley et al., 2018), our systematic review exclusively incorporates literature published after 2016, a factor that may introduce potential bias and compromise the accuracy of our conclusions. Although the number of studies we included is substantial (51 studies), most of the literature is of low to moderate quality, with only 13.4% comprising high-quality studies. This increased likelihood of bias could impact the validity of our findings. Furthermore, the sample size of the studies included in our review ranges from 1 to 24 participants. Generally, for psychometric research, an ideal sample size should exceed 50 (Mokkink et al., 2010). This relatively small sample size may also contribute to additional bias in our conclusions and subsequent misinterpretation.

Moreover, the heterogeneity of included studies represents another potential source of bias. Currently, there is no standardized IMU measurement process. Out of the 51 studies included in this review, there is no consensus on the standard system calibration methods, data fusion algorithms, and biomechanical models utilized. In addition, different studies use a variety of result parameters (ICC, *r*, RMSE, LoA, etc.), making it challenging to extract high-quality data and conduct accurate meta-analysis.

In this study, the meta-analysis employed a methodology similar to that used by Zeng et al. (2022) for data inclusion. This approach entailed performing meta-analysis on the results of IMU measurement validity across different tasks within the same study. The primary benefit of this method is its ability to incorporate a larger sample size, thereby enhancing the efficiency of statistical test. However, this method is with potential drawbacks. The repeated inclusion of results from a single study could obscure the heterogeneity that exists between different studies. Furthermore, if a particular study utilizes a more reliable fusion algorithm or IMUs with lower measurement error, the repeated inclusion of data under the same experimental conditions might lead to an overestimation of the IMU’s measurement validity. Conversely, studies with less reliable algorithms or higher measurement errors could lead to an underestimation of validity. Therefore, while this approach allows for a broader inclusion of data, it also introduces the risk of bias in the overall assessment of IMU measurement validity.

## 5. Conclusions

The findings of this systematic review suggested that IMUs are a promising tool for measuring the RoM of the upper extremity, with good to excellent agreement and very strong correlation compared to OMC. However, caution is advised when using IMUs to measure certain joint movements, such as shoulder internal–external rotation and wrist ulnar-radial deviation. Subgroup analysis revealed that IMUs were less valid than OMC in measuring complex upper-limb movements across multiple planes of motion. To facilitate practical application, further research and standardization are needed to establish guidelines for sensor placement, calibration methods, and data fusion algorithms.

## Supporting information

Li et al. supplementary material 1 Li et al. supplementary material

Li et al. supplementary material 2 Li et al. supplementary material

Li et al. supplementary material 3 Li et al. supplementary material

## Data Availability

All data and material reported in this systematic review are from peer-reviewed publications.

## Abbreviations

Abd: abduction
Add: adduction
CMC: coefficient of multiple correlation
ER: external rotation
Ext: extension
Flex: flexion
HQ: high quality
ICC: intraclass correlation coefficient
IMUs: inertial measurement units
IR: internal rotation
LoA: limit of agreement
LQ: low quality
MQ: moderate quality
OMC: marker-based motion capture system
PRISMA: Preferred Reporting Items for Systematic Reviews and Meta-Analyses
Pron: pronation
RMSE: root mean square error
SD: standard deviation
Supi: supination
VLQ: very low quality.

## Supplementary material

The supplementary material for this article can be found at https://doi.org/10.1017/wtc.2024.6.
