# Supplementary material for: Concurrent validity of inertial measurement units in range of motion measurements of upper extremity: A systematic review and meta-analysis
Source: Wearable Technol. 2024 Oct 4;5:e11. doi: 10.1017/wtc.2024.6 (PMC11503723; doi:10.1017/wtc.2024.6)
Supplement: Li et al. supplementary material 1 — Li et al. supplementary material [file S2631717624000069sup001.docx]

**Complete Inclusion and Exclusion Criteria**

**Inclusion:**

1. Evaluated the validity of inertial measurement units (IMUs),

—Concurrent validity: reported the results of simultaneous measurement by IMUs and marker-based motion capture systems.

1. Measured and reported specific upper extremity range of motion (RoM) results,

— Included upper extremity RoM outcomes: shoulder, elbow and wrist joints.

1. Compared the measurements captured by IMUs to the marker-based motion capture systems,
2. Assessed human beings,
3. Published in English.

**Exclusion:**

1. No relevant outcomes,

— Only reported velocity, acceleration; or no validity related outcomes: ICC, Pearson’s r, RMSE, mean ± SD, MAD, CMC, LoA, and so on.

1. No comparison with standard marker-based motion capture systems,

— Excluded Microsoft Kinect system, RGB-D system, and so on.

1. Only assessed lower limb motion,
2. Only assessed unnatural human motion,

— Excluded Robotic assistant motion and exoskeleton devices.

1. Animal model studies,
2. Only assessed children and infants,
3. No research studies or no full text,

— literature review papers; letters to the editor; abstracts published in conference proceedings.

1. Published in other languages.
