# Supplementary material for: Concurrent validity of inertial measurement units in range of motion measurements of upper extremity: A systematic review and meta-analysis
Source: Wearable Technol. 2024 Oct 4;5:e11. doi: 10.1017/wtc.2024.6 (PMC11503723; doi:10.1017/wtc.2024.6)
Supplement: Li et al. supplementary material 2 — Li et al. supplementary material [file S2631717624000069sup002.docx]

**Critical Appraisal of Study Design for Psychometric Articles**

**Rubrics**

To decide which score to provide for each item on a quality checklist, read the following descriptors. Pick the descriptor that sounds most like the study being evaluated with respect to a given item. If there is no documentation of an action, treat it as not done.

Referred from:

1. Kobsar D, Charlton JM, Tse CTF, Esculier JF, Graffos A, Krowchuk NM, et al. Validity and reliability of wearable inertial sensors in healthy adult walking: a systematic review and meta-analysis. J Neuroeng Rehabil. 2020;17(1):62.

| **Question** | **Score** | **Description** |
| --- | --- | --- |
| **Study Question** | | |
| 1  Background & Research Question | 2 | 1. Performed a thorough literature review, indicating what is currently known about the psychometric properties of the instruments or tests under study from previous research studies.  2. Presented a critical and unbiased view of the current state of knowledge.  3. Indicated how the current research question evolves from a gap in the current knowledge base.  4. Established a research question based on the above. |
|  | 1 | All of the above criteria were not fulfilled (little reference to previous research and present gaps in knowledge), but a clear rationale was provided for the research question. |
|  | 0 | A foundation for the current research question was not clear, and the rationale was not founded on previous literature |
| **Study Design** | | |
| 2  Subjects | 2 | Provide specific inclusion/exclusion criteria more than simply “healthy” or “control”, provide age range/cut-off for recruitment, report demographic data (age, sex, body composition), and recruitment setting/context. |
|  | 1 | No specific inclusion/exclusion criteria other than “healthy” or “control”, no a priori age definition, and no information on setting/context, but does report demographic data (age, sex, body composition). |
|  | 0 | No specific inclusion/exclusion criteria other than “healthy” or “control”, no a priori age definition, no information on setting/context, and is missing some demographic data (age, sex, or body composition). |
| 3  Objective/  Hypothesis | 2 | Authors identified objectives/hypotheses, which described the specific type of validity (criterion) evaluated against a defined gold-standard (intro). Include an a priori hypothesis/definition of level of the validity that would be expected/acceptable. This information is provided before the methods/reporting benchmarks. |
|  | 1 | Authors identified objectives/hypotheses, which described the specific type of validity (criterion) evaluated against a defined gold-standard (intro). Did NOT include an a priori hypothesis/definition of level of the validity that would be expected/acceptable. |
|  | 0 | Authors did not identify specific types of validity under evaluation. For example, “The purpose of this study was to investigate validity of XXX from a wearable sensor.” can be rated as zero if no further detail on the types of reliability and validity or the nature of specific objectives/hypotheses are provided. |
| 4  Scope/  Design | 2 | Authors provided detailed information on the how the gold-standard was to be collected with the device being tested. |
|  | 1 | Limited information was provided on how the specific type of validity was assessed. |
|  | 0 | No information was provided on how the specific type of validity was assessed. |
| 5  Sample | 2 | Authors performed a sample size calculation and obtained their recruitment targets. Post-hoc power analyses and/or confidence intervals (CI) confirm that the sample size was sufficient to define relatively precise estimates of validity. |
|  | 1 | The authors provided a rationale for the number of subjects included in the study but did not present specific sample size calculations or post-hoc power analyses. |
|  | 0 | Size of the sample was not rationalized or is clearly underpowered. |
| 6  Retention | 2 | Ninety percent or more of the patients enrolled for study were reevaluated. |
|  | 1 | More than 70% of the patients eligible for study were reevaluated. |
|  | 0 | Less than 70% of the patients eligible for study were reevaluated. |
| **Measurements** | | |
| 7  Sensor | 2 | The authors provided detailed information that outlines the measurement device (i.e., inertial sensor) and specific procedures for data collections. This information needs to consist of: i) name, manufacturer of sensor, and sampling rate, ii) specifics on placement, and iii) sensor calibration procedures (before or on participant). |
|  | 1 | Device is referenced with moderate description/information (2 complete categories out of 3) |
|  | 0 | Minimal description of device is provided and without appropriate references. (0 or 1 categories out of 3) |
| 8  Protocol | 2 | The authors provided detailed information that outlines the task to be assessed. This information needs to consist of: i) calibration of sensor and marker-based motion capture system, ii) details of upper limb movement patterns (involved joints, movement axes and/or planes, etc.), iii) setting (lab or daily life), and iv) information on how devices were collected simultaneously. |
|  | 1 | No obvious sources of bias in how tests were performed/administered, but minimal attention or description of the extent to which the above standards were maintained. Specifically, 3/4 items were reported. |
|  | 0 | No description of the extent to which the above standards were maintained or an obvious source of bias in data collection methods. Less than 3 items were reported. |
| **Analyses** | | |
| 9  Organization | 2 | Authors clearly defined which specific analyses were conducted for each of the stated specific hypotheses of the study. This may be accomplished through organization of the results under specific subheadings, or by demarcating which analyses addressed specific psychometric properties. Data were presented for each objective/hypothesis. |
|  | 1 | Data were presented for each specific objective/hypothesis, but authors did not clearly link these analyses to the defined objective/hypothesis. |
|  | 0 | Data were not presented for each objective/hypothesis or psychometric property outlined in the purposes or methods. |
| 10  Statistical Analyses | 2 | Appropriate statistical tests were conducted:  ICCs, Pearson’s r or provided Mean ± SD |
|  | 1 | Appropriate statistical tests were used in some instances, but suboptimal choices were made in other analyses:  RMSE, Limits of agreement (Bland-Altman plot), CMC, or Spearman. |
|  | 0 | None of the above outcomes:  Only reporting other outcomes (coefficient of variation, raw data, etc.) would be a 0. |
| 11  Estimates of Variance | 2 | For key indicators such as validity coefficients indices, at least 2 of the following were presented:  1. Appropriate confidence intervals (CI)  2. Comparison to appropriate benchmarks or standards (reference or rationale provided)  3. SEM  Correlation matrices for validity analysis may not require that each individual correlation be presented with its associated CI; however, CIs and benchmarks should be used according to standards for this type of analysis. |
|  | 1 | Either CIs or appropriate benchmarks were used, but not both. |
|  | 0 | Benchmarks or CIs were either used inappropriately or not included. |
| **Recommendations** | | |
| 12  Conclusion/  Recommendations | 2 | Authors made specific conclusions and clinical recommendations that were clearly related to the specific objectives/hypotheses stated at the beginning of the study and supported by the data presented. |
|  | 1 | Authors made conclusions and clinical recommendations that were general, but basically supported by the study data, OR authors made conclusions and clinical recommendations for only some of the study objective/hypotheses. |
|  | 0 | Authors made vague conclusions without any clinical recommendations, and conclusions OR recommendations contradicted the actual data presented. |
