# Supplementary material for: Concurrent validity of inertial measurement units in range of motion measurements of upper extremity: A systematic review and meta-analysis
Source: Wearable Technol. 2024 Oct 4;5:e11. doi: 10.1017/wtc.2024.6 (PMC11503723; doi:10.1017/wtc.2024.6)
Supplement: Li et al. supplementary material 3 — Li et al. supplementary material [file S2631717624000069sup003.docx]

**Quantitative data summary of included literature**

| **Study information** | | **Sample size(N)** | **Results** | | | | | |
| --- | --- | --- | --- | --- | --- | --- | --- | --- |
| **Author** | **Year** |  | **Mean (SD)/°** | **ICC/r** | **RMSE(SD)/°** | **LOA** | **Bias (°)** | **Others** |
| Abbasi-Kesbi, et al. | 2018 | 1 | N/A | N/A | Movement1:  Complementary filter:  Elbow:  Φ:0.92(0.89)  Ψ:1.61(1.57)  Θ:0.91(0.87)  Wrist:  Φ:0.88(0.83)  Ψ:1.63(1.56)  Θ:0.90(0.85)  Modified complementary filer:  Elbow:  Φ:0.84(0.82)  Ψ:1.59(1.56)  Θ:0.73(0.71)  Wrist:  Φ:0.82(0.79)  Ψ:1.61(1.55)  Θ:0.75(0.71)  Movement2:  Complementary filter:  Elbow:  Φ:0.84(0.81)  Ψ:1.53(1.5)  Θ:1.05(1.01)  Wrist:  Φ:0.98(0.94)  Ψ:1.65(1.62)  Θ:1.01(0.97)  Modified complementary filer:  Elbow:  Φ:0.66(0.65)  Ψ:1.5(1.49)  Θ:0.98(0.96)  Wrist:  Φ:0.78(0.75)  Ψ:1.64(1.62)  Θ:0.92(0.89) | N/A | N/A | N/A |
| Alarcon-Aldana, et al. | 2022 | 1 | N/A | N/A | 3.82  3.46 | N/A | N/A | N/A |
| Barreto, et al. | 2021 | 10 | N/A | N/A | Shoulder:  Abd./Add.:9.86(3.90)  Flex./Ext.:12.57(3.42)  IR/ER:8.46(4.59)  Elbow:  Flex./Ext.:4.20(1.38)  Wrist:  Flex./Ext.:6.91(2.00) | N/A | N/A | CMC  Shoulder:  Abd./Add.:0.88(0.12)  Flex./Ext.:0.99(0.00)  IR/ER:0.98(0.01)  Elbow:  Flex./Ext.:0.96(0.04)  Wrist:  Flex./Ext.:0.99(0.00) |
| Bessone, et al. | 2019 | 14 | Shoulder:  Flex./Ext.:  IMU:131.3(20.6)  OMC:123.5(16.2)  Abd./Add:  IMU:96.3(8.6)  OMC:89.4(11.7)  Elbow:  Flex./Ext.  IMU:142.2(17.1)  OMC:120(9.5)  Wrist:  Flex./Ext.  IMU:123.1(18.6)  OMC:104.3(21.2)  U/R devi.:  IMU:51.6(12.2)  OMC:39.1(8.4) | Shoulder:  Abd./Add.:0.796  Flex./Ext.:0.782  Elbow:  Flex./Ext.:0.655  Wrist:  Flex./Ext.:0.872  Ulnar deviation:0.26 | Shoulder:  Abd./Add.:10.9  Flex./Ext.:14.6  Elbow:  Flex./Ext.:27.1  Wrist:  Flex./Ext.:17.6  Ulnar deviation:21.2 | N/A | Shoulder:  Abd./Add.:-7.0±17.1  Flex./Ext.:-7.8±25.0  Elbow:  Flex./Ext.:-22.1±32.0  Wrist:  Flex./Ext.:-18.8±20.3  Ulnar deviation:-12.4±25.3 | N/A |
| Boddy, et al. | 2019 | 10 | Shoulder:  IR/ER:  Fastball:  IMU:156(5)  OMC:167(2)  Off-speed:  IMU:157(3)  OMC:168(3) | Shoulder:  IR/ER:  Fastball:0.71  Off-speed:0.784 | N/A | N/A | N/A | N/A |
| Callejas-Cuervo, et al. | 2016 | 1 | N/A | N/A | 2.44  2.12  2.28 | N/A | N/A | N/A |
| Camp. et al. | 2021 | 10 | N/A | N/A | 1.2-8.1 | N/A | N/A | N/A |
| Chan, et al. | 2022 | 19 | Shoulder:  Flex.:  IMU:92.4(6.65)  OMC:87.2(7.25)  Ext.:  IMU:34(7.43)  OMC:36.58(7.84)  Abd./Add:  IMU:92.42(4.48)  OMC:84.87(6.19)  IR/ER:  IMU:58.56(17.8)  OMC:52.44(15.2) | N/A | Flex.:7.62  Ext.:5.04  ER.:10.08  Abd.:8.75 | Lower LOA:  Flex.:-16.41[-21.2,-11.61]  Ext.:-2.29[-10.01,-6.15]  ER.:-22.27[-29.41,-15.13]  Abd.:-16.45[-20.26,-12.64]  Upper LOA:  Flex.:6.0[1.21,10.8]  Ext.:11.31[7.45,15.17]  ER.:10.03[2.09,17.17]  Abd.:1.35[-2.46,5.16] | Flex.-5.20  Ext.:2.58  ER.:-6.12  Abd.:-7.55 | R^2^  Flex.0.44  Ext.:0.69  ER.:0.79  Abd.:0.46 |
| Chapman, et al. | 2019 | 10 | N/A | N/A | N/A | N/A | N/A | R^2^  Flex.:0.96068  Abd.:0.96482  IR/ER:0.89585 |
| Chen, et al. | 2020 | 14 | N/A | N/A | Arm elevation:  Slow transfer rate:  Comp. w/o bias:1.9(1.4)  Comp. w/ bias:1.4(0.9)  KF:1.7(1.2)  KF w/ bias:1.0(0.6)  Medium transfer rate:  Comp. w/o bias:2.1(1.2)  Comp. w/ bias:1.6(0.9)  KF:1.9(1.1)  KF w/ bias:1.1(0.6)  Fast transfer rate:  Comp. w/o bias:2.4(1.3)  Comp. w/ bias:1.6(0.8)  KF:2.1(1.2)  KF w/ bias:1.2(0.5) | N/A | N/A | N/A |
| Choo, et al. | 2022 | 7 | N/A | N/A | Stationary walk  Elbow  Flex./Ext.:3.40(2.15)  Shoulder  Flex./Ext.:1.90(0.80)  Abd./Add.:7.14(2.97)  Distance walk  Elbow  Flex./Ext.:2.04(1.48)  Shoulder  Flex./Ext.:1.12(0.65)  Abd./Add.:5.36(3.16)  Stationary jog  Elbow  Flex./Ext.:3.89(2.96)  Shoulder  Flex./Ext.:1.94(1.53)  Abd./Add.:5.97(3.80)  Distance jog  Elbow  Flex./Ext.:1.92(1.00)  Shoulder  Flex./Ext.:1.78(1.16)  Abd./Add.:5.70(2.57)  Stationary floorball wrist shot  Elbow  Flex./Ext.:2.81(2.18)  Shoulder  Flex./Ext.:2.23(1.97)  Abd./Add.:11.85(10.24)  Moving wrist shot  Elbow  Flex./Ext.:3.20(1.75)  Shoulder  Flex./Ext.:1.99(1.12)  Abd./Add.:15.15(9.32) | Stationary walk  Elbow  Flex./Ext.:-16.2[-42.55,10.15]  Shoulder  Flex./Ext.:4.04[-10.42,18.50]  Abd./Add.:9.07[-20.59,38.73]  Distance walk  Elbow  Flex./Ext.:-21.17[-35.47,-6.88]  Shoulder  Flex./Ext.:7.26[-3.49,18.01]  Abd./Add.:11.10[-22.63,44.82]  Stationary jog  Elbow  Flex./Ext.:-15.06[-68.01,37.90]  Shoulder  Flex./Ext.:2.61[-11.87,17.09]  Abd./Add.:12.16[-15.39,39.72]  Distance jog  Elbow  Flex./Ext.:-12.22[-28.02,3.57]  Shoulder  Flex./Ext.:3.54[-8.31,15.39]  Abd./Add.:9.80[-22.24,41.85]Stationary floorball wrist shot  Elbow  Flex./Ext.:-20.34[-40.53,-0.15]  Shoulder  Flex./Ext.:1.92[-23.74,27.58]  Abd./Add.:-7.52[-69.77,54.72]  Moving wrist shot  Elbow  Flex./Ext.:-20.62[-41.82,0.57]  Shoulder  Flex./Ext.:-1.42[-26.81,23.98]  Abd./Add.:2.88[-61.27,67.03] | N/A | R^2^  Stationary walk  Elbow  Flex./Ext.:0.96  Shoulder  Flex./Ext.:0.80  Abd./Add.:0.77  Distance walk  Elbow  Flex./Ext.:0.91  Shoulder  Flex./Ext.:0.59  Abd./Add.:0.66  Stationary jog  Elbow  Flex./Ext.:0.85  Shoulder  Flex./Ext.:0.82  Abd./Add.:0.79  Distance jog  Elbow  Flex./Ext.:0.94  Shoulder  Flex./Ext.:0.88  Abd./Add.:0.87  Stationary floorball wrist shot  Elbow  Flex./Ext.:0.84  Shoulder  Flex./Ext.:0.76  Abd./Add.:0.58  Moving wrist shot  Elbow  Flex./Ext.:0.85  Shoulder  Flex./Ext.:0.91  Abd./Add.:0.50 |
| Contreras-Gonzalez, et al. | 2020 | 9 | N/A | N/A | Abd./Add.:1.89  Flex./Ext.:2.30  Horiz. Add.:2.23  Rotation:1.51 | N/A | N/A | N/A |
| Digo, et al. | 2022 | 6 | N/A | N/A | Shoulder elevation:2.4(1.1)  Elbow Flex./Ext.:3.5(1.1) | N/A | N/A | N/A |
| Dufour, et al. | 2021 | 11 | N/A | N/A | Shoulder elevation:  Low difficulty:2.18(0.85)  High difficulty:2.06(1.23) | N/A | N/A | Accuracy  Shoulder elevation:  Low difficulty:98.7%(0.5%)  High difficulty:98.7%(0.7%) |
| Erika, et al. | 2019 | 20 | N/A | N/A | PSUP:7.34  THFF:2.12  FTAP:1.51 | N/A | N/A | R2  PSUP:0.949  THFF:0.987  FTAP:0.927 |
| Ertzgaard, et al. | 2016 | 10 | N/A | Cone  Shoulder:  Flex./Ext.0.952  Abd./Add.:0.879  ER/IR:0.943  Elbow:  Flex.:0.984  Prona./Supina.:0.682  Throw  Shoulder:  Flex./Ext.0.975  Abd./Add.:0.817  ER/IR:0.905  Elbow:  Flex.:0.845  Prona./Supina.:0.783  CT1  Shoulder:  Flex./Ext.0.902  Abd./Add.:0.949  ER/IR:0.799  Elbow:  Flex.:0.914  Prona./Supina.:0.792  CT2  Shoulder:  Flex./Ext.0.943  Abd./Add.:0.946  ER/IR:0.768  Elbow:  Flex.:0.888  Prona./Supina.:0.808 | N/A | N/A | N/A | N/A |
| Esfahani, et al. | 2018 | 1 | N/A | N/A | Flex.(45)  Roll angle:3.21  Pitch angle:2.84  Yaw angle:3.53  Flex.(90)  Roll angle:3.1  Pitch angle:2.5  Yaw angle:3.38  Flex.(135)  Roll angle:2.69  Pitch angle:2.09  Yaw angle:2.97  Flex.(180)  Roll angle:2.78  Pitch angle:2.43  Yaw angle:3.1  Ext.  Roll angle:3.1  Pitch angle:2.15  Yaw angle:2.92  Abd.(45)  Roll angle:2.11  Pitch angle:1.75  Yaw angle:3.73  Abd.(90)  Roll angle:2.79  Pitch angle:3.04  Yaw angle:3  Abd.(135)  Roll angle:3.5  Pitch angle:2.34  Yaw angle:3.74  Abd.(180)  Roll angle:3.5  Pitch angle:2.79  Yaw angle:3.61  IR/ER  Roll angle:2.94  Pitch angle:3.15  Yaw angle:3.86 | N/A | N/A | R2  Flex.(45)  Roll angle:0.96  Pitch angle:0.97  Yaw angle:0.94  Flex.(90)  Roll angle:0.99  Pitch angle:0.99  Yaw angle:0.97  Flex.(135)  Roll angle:0.98  Pitch angle:0.96  Yaw angle:0.95  Flex.(180)  Roll angle:0.97  Pitch angle:0.98  Yaw angle:0.95  Ext.  Roll angle:0.99  Pitch angle:0.97  Yaw angle:0.97  Abd.(45)  Roll angle:0.96  Pitch angle:0.95  Yaw angle:0.95  Abd.(90)  Roll angle:0.97  Pitch angle:0.95  Yaw angle:0.97  Abd.(135)  Roll angle:0.99  Pitch angle:0.97  Yaw angle:0.95  Abd.(180)  Roll angle:0.97  Pitch angle:0.95  Yaw angle:0.98  IR/ER  Roll angle:0.96  Pitch angle:0.93  Yaw angle:0.94 |
| Fantozzi, et al. | 2016 | 8 | N/A | N/A | Front-crawl swimming  Shoulder  Flex./Ext.:5(4,8)  Abd./Add.:7(5,9)  IR/ER:7(5.8)  Elbow  Flex./Ext.:15(12,17)  Prona./Supin:10(7,11)  Wrist  Flex./Ext.:5(4,5)  Radial–ulnar:3(2,4)  Breaststroke swimming  Shoulder  Abd./Add.:5(3,6)  IR/ER:3(3,4)  Elbow  Flex./Ext.:8(5,10)  Prona./Supin:6(5,10)  Wrist  Flex./Ext.:5(4,7)  Radial–ulnar:4(3,5) | N/A | N/A | CMC  Front-crawl swimming  Shoulder  Flex./Ext.:0.99  Abd./Add.:0.97  IR/ER:0.99  Elbow  Flex./Ext.:0.95  Prona./Supin:0.93  Wrist  Flex./Ext.:0.95  Radial–ulnar:0.9  Breaststroke swimming  Shoulder  Abd./Add.:0.99  IR/ER:0.99  Elbow  Flex./Ext.:0.98  Prona./Supin:0.97  Wrist  Flex./Ext.:0.98  Radial–ulnar:0.93 |
| Fischer, et al. | 2021 | 10 | Flex./Ext.:  Marker on skin:  IMU:101.4(29)  OMC:99.8(14.5)  Marker on sensor  IMU:101.4(29)  OMC:122.5(27.1)  U/R  Marker on skin  IMU:59.1(12.5)  OMC:47.7  Marker on sensor  IMU:59.1(12.5)  IMU:57.3(8.7) | N/A | Flex./Ext.  IMU vs. OMC1:8.3(2.5)  IMU.vs. OMC2:15.3(4.7)  Radial-ulnar  MU vs. OMC1:9.9(3.2)  IMU.vs. OMC2:8.2(2.3) | N/A | N/A | MAD  Flex./Ext.  IMU vs. OMC1:8.2(4.0)  IMU.vs. OMC2:23.0(8.8)  Radial-ulnar  MU vs. OMC1:17.3(8.0)  IMU.vs. OMC2:11.1(5.1) |
| Goreham, et al. | 2022 | 15 | N/A | S-EFE:0.44  S-SAA:0.14  S-SFE:0.13  S-SIER:0.33  HBP-EFE:0.64  HBP-SAA:0.47  HBP-SFE:0.40  HBP-SIER:0.16  HCS-EFE:0.25  HCS-SAA:0.23  HCS-SFE:0.39  HCS-SIER:0.18  HTH-EFE:0.47  HTH-SAA:0.85  HTH-SFE:0.21  HTH-SIER:0.36 | N/A | N/A | N/A | N/A |
| Guignard, et al. | 2021 | 1 | N/A | Right elbow  Flex./Ext.:0.979[0.978,0.980]   1. S:0.979[0.978,0.980]   Walking:0.981[0.980,0.981]  Crawl:0.986[0.985,0.987]  Rowing:0.985[0.985,0.986]  Left elbow  Flex./Ext.:0.897[0.893,0.900]  P-S:0.988[0.988,0.989]  Walking:0.949[0.948,0.951]  Crawl:0.866[0.858,0.873]  Rowing:0.946[0.944,0.948] | Right elbow  Flex./Ext.:8.23  P-S:9.36  Walking:5.98  Crawl:5.60  Rowing:6.53  Left elbow  Flex./Ext.:16.28  P-S:7.24  Walking:9.76  Crawl:13.38  Rowing:12.46 | N/A | N/A | N/A |
| Henschke, et al. | 2022 | 24 | Shoulder:  Flex./Ext.  IMU:170.4(21.4)  OMC:195.1(18.9)  Abd./Add.  IMU:155.2(19.9)  OMC:194(24.9)  IR/ER:  IMU:72.2(15.6)  OMC:122.9(36.9) | Abd.:0.048  Add.:0.534  H-F:0.727  H-E:0.296  V-F:0.522  V-E:0.369  ER:0.040  IR:0.208 | Abd.:12.2  Add.:12.8  H-F:13.0  H-E:9.7  V-F:14.0  V-E:17.9  ER:10.7  IR:10.4 | Abd.:[11.9,84.5]  Add.:[-31.8,24.8]  H-F:[-23.5,26.0]  H-E:[-22.4,28.4]  V-F:[-16.0,39.2]  V-E:[-19.0,49.5]  ER:[-7.6,59.3]  IR:[10.1,54.1] | Abd.:48.2  Add.:-3.5  H-F:1.2  H-E:3.0  V-F:11.6  V-E:15.3  ER:25.9  IR:32.1 | N/A |
| Hubaut, et al. | 2022 | 10 | N/A | N/A | N/A | Yaw angle;[-3.180,3.628]  Pitch angle:[-2.397,2.676]  Roll angle:[-4.474,2.745] | Yaw angle:0.224(2.1)  Pitch angle:0.139(1.599)  Roll angle:-0.864(0.237) | R2  Yaw angle:0.976(0.028)  Pitch angle:0.924(0.092)  Roll angle:0.951(0.084) |
| Humadi, et al. | 2021 | 10 | N/A | N/A | Packing  Elbow Flex.  Right:4.5(4.7)  Left:6.4(6.5)  Shoulder  Flex.  Right:5.2(2.9)  Left:5.1(3.7)  Add.  Right:4.2(3.7)  Left:3.47(2.2)  Rot.  Right:10.8(9.5)  Left:7.9(5.9)  Package inspection  Elbow Flex.  Right:7.0(3.0)  Left:7.9(3.6)  Shoulder  Flex.  Right:8.0(5.1)  Left:7.4(5.4)  Add.  Right:4.1(2.2)  Left:6.1(6.2)  Rot.  Right:6.8(6.8)  Left:7.9(5.9)  Reaching object  Elbow Flex.  Right:8.3(5.4)  Left:9.9(2.9)  Shoulder  Flex.  Right:8.5(6.3)  Left:8.2(6.0)  Add.  Right:4.9(3.2)  Left:5.0(3.2)  Rot.  Right7.6(18.9)  Left:5.0(9.3) | N/A | N/A | N/A |
| Laidig, et al. | 2017 | 1 | N/A | N/A | N/A | N/A | N/A | Dtand. Dev.  Flex./Ext.:3.43  Pron./Supin.:6.63 |
| Ligorio, et al. | 2017 | 15 | N/A | N/A | Elbow  Flex./Ext  Less than 4 | N/A | N/A | N/A |
| Ligorio, et al. | 2020 | 10 | N/A | Shoulder  Flex./Ext.:0.96  Abd./Add.:0.93  IR.ER:0.92  Elbow  Flex./Ext.:0.95  Carrying angle:0.91  Prona./Supina.:0.94  Wrist  Flex.Ext.:0.98  Abd./Add.:0.97  IR/ER:0.97 | N/A | N/A | N/A | N/A |
| Lin, et al. | 2021 | 13 | N/A | N/A | Roll angle  Flex.:2.87±0.85  Abd.:4.40±1.14  Ext.:4.14±1.31  ER:3.08±2.63  IR:3.01±1.29  Pitch angle  Flex.:1.62±0.77  Abd.:1.11±0.35  Ext.:1.64±0.63  ER:1.34±0.38  IR:1.40±0.65  Yaw angle  Flex.:3.09±1.08  Abd.:4.88±1.70  Ext.:4.91±1.79  ER:3.36±2.56  IR:2.81±1.39 | N/A | N/A | SEM  Flex.:3.62  Abd.:2.51  Ext.:0.95  ER:1.86  IR:1.91 |
| Marta, et al. | 2020 | 2 | N/A | Flex./Ext.:0.994  Abd./Add.:0.991 | Flex./Ext.:5.6  Abd./Add.:4.7 | N/A | N/A | N/A |
| Mavor, et al. | 2020 | 20 | N/A | N/A | Right shoulder  Flex./Ext.:11.0(6.46)  Abd./Add.:8.79(3.16)  Axial Rot,11.5(6.62)  Left shoulder  Flex./Ext.:13.4(8.44)  Abd./Add.:10.6(3.52)  Axial Rot.:15.1(10.9)  Right elbow  Flex./Ext.:10.6(4.45)  Abd./Add.:11.8(4.06)  Axial Rot.:16.7(7.25)  Left elbow  Flex./Ext.:11.3(5.36)  Abd./Add.:11.5(3.85)  Axial Rot.:15.2(6.72) | N/A | N/A | N/A |
| Mihcin,et al. | 2019 | 1 | N/A | N/A | Shoulder flex.:0.66  Elbow flex.:0.51 | N/A | N/A | N/A |
| Morrow, et al. | 2017 | 6 | N/A | N/A | Shoulder elevation:6.8(2.7)  Elbow flex.:8.2(2.8) | N/A | N/A | N/A |
| Muller, et al. | 2017 | 1 | N/A | N/A | Proposed algorithm  Flex./Ext.:2.7  P-S:3.8  Manual alignment  Flex./Ext.:3.8  P-S:8.7 | N/A | N/A | N/A |
| Ohberg, et al. | 2019 | 20 | N/A | N/A | N/A | N/A | Finger to nose task:  0.25-1.1  Drinking task:  0.45-0.84 | N/A |
| Pedro, et al. | 2021 | 18 | N/A | N/A | Shoulder  Flex./Ext.:6.1  Abd./Add.:3.5  IR/ER:4.1  Elbow  Flex./Ext.:1.5  Prona./Supina.:13.1  Wrist  Flex./Ext.:6.3 | N/A | N/A | CMC  Shoulder  Flex./Ext.:0.95  Abd./Add.:0.89  IR/ER:0.99  Elbow  Flex./Ext.:0.99  Prona./Supina.:0.79  Wrist  Flex./Ext.:0.98 |
| Picerno, et al. | 2019 | 14 | Shoulder:  IR/ER:  IMU:42.3(8.1)  OMC:44.9(8.9)  Elbow:  Flex./Ext.  IMU:108.1(16.2)  OMC:109.3(13.9)  P/S  IMU:117.3(15.5)  OMC:119.5(14.1) | N/A | Shoulder  Axial rotation:1.8±1.4  Arm elevation  Frontal plane:4.4±4.1  Scapular plane:2.5±1.7  Sagittal plane:2.3±2.5  Elbow  Flex./Ext.:1.9±2.6  P-S:2.9±1.6 | Shoulder  Axial rotation:-2.6[-9.0,3.9]  Arm elevation  Frontal plane:-11.7[-24.7,1.3]  Scapular plane:-8.6[-19.9,2.7]  Sagittal plane:-1.9[-13.0,9.2]  Elbow  Flex./Ext.:-1.2[-10.6,8.3]  P-S:-2.2[-15.0,10.7] | N/A | R2  Shoulder  Axial rotation:0.995±0.009  Arm elevation  Frontal plane:0.997±0.003  Scapular plane:0.997±0.003  Sagittal plane:0.997±0.003  Elbow  Flex./Ext.:0.999±0.002  P-S:0.997±0.004 |
| Poitras, et al. | 2019 | 16 | N/A | Flex.  60 degree:0.968  90 degree:0.998  120 degree:0.997  Abd.  60 degree:0.998  90 degree:0.999  120 degree:0.999 | Flex.  60 degree:5.17(2.81)  90 degree:4.67(2.95)  120 degree:6.21(3.90)  Abd.  60 degree:2.77(1.28)  90 degree:3.75(2.86)  120 degree:4.92(3.02) | N/A | N/A | N/A |
| Robert-Lachaine, et al. | 2020 | 5 | N/A | N/A | Shoulder  X axis:6.0  Z axis:4.9  Y axis:10.3  Elbow  Z axis:13.4  X axis:9.7  Y axis:12.3  Wrist  Z axis:8.9  X axis:6.6  Y axis:9.6 | Shoulder  X axis:-0.6±9.9  Z axis:1.8±8.3  Y axis:-4.3±14.0  Elbow  Z axis:-11.7±9.5  X axis:0.1±14.1  Y axis:8.4±15.2  Wrist  Z axis:5.6±9.6  X axis:3.9±8.8  Y axis:-0.9±11.8 | N/A | CMC  Shoulder  X axis:0.98  Z axis:0.98  Y axis:0.85  Elbow  Z axis:0.91  X axis:0.85  Y axis:0.87  Wrist  Z axis:0.81  X axis:0.78  Y axis:0.76 |
| Robert-Lachaine, et al. | 2016 | 12 | N/A | N/A | Shoulder  X axis:2.9  Z axis:3.0  Y axis:2.5  Elbow  Z axis:2.9  X axis:2.0  Y axis:2.6  Wrist  Z axis:3.8  X axis:2.8  Y axis:3.6 | Shoulder  X axis:-0.1±5.2  Z axis:-0.9±4.2  Y axis:-0.7±3.8  Elbow  Z axis:0.3±5.2  X axis:0.3±3.7  Y axis:0.6±4.8  Wrist  Z axis:-1.0±6.9  X axis:-0.4±5.1  Y axis:-1.3±5.9 | N/A | CMC  Shoulder  X axis:1.00  Z axis:0.99  Y axis:0.99  Elbow  Z axis:0.99  X axis:0.98  Y axis:0.99  Wrist  Z axis:0.96  X axis:0.95  Y axis:0.92 |
| Robert-Lachaine, et al. | 2017 | 12 | N/A | N/A | N/A | N/A | N/A | SEM  Shoulder  X axis:1.5  Z axis:1.5  Y axis:2.7  Elbow  Z axis:2.1  X axis:1.3  Y axis:3.5  Wrist  Z axis:1.8  X axis:1.2  Y axis:1.8 |
| Ruiz-Malagon, et al. | 2022 | 15 | N/A | N/A | 100Hz  Flex./Ext.:8.66  Pron./Sup.:8.53  200Hz  Flex./Ext.:7.53  Pron./Sup.:7.66  300Hz  Flex./Ext.:5.76  Pron./Sup.:6.66 | N/A | N/A | Lin’s CCC  100Hz  Flex./Ext.:0.81  Pron./Sup.:0.83  200Hz  Flex./Ext.:0.85  Pron./Sup.:0.86  300Hz  Flex./Ext.:0.89  Pron./Sup.:0.86 |
| Schall, et al. | 2016 | 6 | N/A | N/A | N/A | N/A | N/A | Slope  Shoulder:  -9.38 |
| Sers, et al. | 2020 | 8 | N/A | N/A | Slow movement:3.2(1.1)  Fast movement:2.9(1.5) | 3.35(-0.68,7.39) | N/A | N/A |
| Shepherd, et al. | 2017 | 4 | N/A | N/A | N/A | N/A | N/A | Error difference  -4.03±1.58 |
| Slade, et al. | 2022 | 5 | N/A | Shoulder  Flex.:0.972  Abd.:0.871  Rot.:0.782  Elbow flex,:0.987  Wrist flex.:0.924 | Shoulder  Flex.:6.9±4.2  Abd.:5.2±0.8  Rot.:7.9±2.6  Elbow flex,:5.2±2.1  Wrist flex.:5.7±2.1 | N/A | N/A | N/A |
| Truppa, et al. | 2021A | 10 | N/A | Shoulder  Flex./Ext.:0.99±0.01  Abd./Add.:0.99±0.01  IR/ER.:0.98±0.01  Elbow  Flex./Ext.:0.97±0.05  Abd./Add.:0.92±0.03  IR/ER.:0.95±0.03  Wrist  Flex./Ext.:0.96±0.05  Abd./Add.:0.99±0.01  IR/ER.:0.91±0.07 | N/A | N/A | N/A | N/A |
| Truppa, et al. | 2021B | 5 | N/A | Shoulder  Flex./Ext.:0.99±0.01  Abd./Add.:0.98±0.02  IR/ER.:0.98±0.02  Elbow  Flex./Ext.:0.88±0.11  Abd./Add.:0.94±0.04  IR/ER.:0.96±0.01  Wrist  Flex./Ext.:0.99±0.01  Abd./Add.:0.86±0.16  IR/ER.:0.88±0.12 | N/A | N/A | N/A | N/A |
| Truppa, et al. | 2022 | 10 | N/A | Shoulder  Flex./Ext.:0.88±0.21  Abd./Add.:0.97±0.03  IR/ER.:0.80±0.30  Elbow  Flex./Ext.:0.96±0.02  Abd./Add.:0.89±0.06  IR/ER.:0.87±0.09  Wrist  Flex./Ext.:0.91±0.20  Abd./Add.:0.96±0.02  IR/ER.:0.91±0.16 | Shoulder  Flex./Ext.:3.20±0.98  Abd./Add.:3.85±2.35  IR/ER.:6.90±4.01  Elbow  Flex./Ext.:2.96±0.95  Abd./Add.:3.49±1.02  IR/ER.:6.79±2.31  Wrist  Flex./Ext.:4.82±2.36  Abd./Add.:4.27±2.17  IR/ER.:8.41±3.73 | N/A | N/A | N/A |
| Wells, et al. | 2019 | 9 | Elbow:  Flex./Ext.:  IMU:144.0(8.2)  OMC:145.2(7.7) | .Elbow:  Flex./Ext.: 0.966 | N/A | N/A | N/A | N/A |
| Wirth, et al. | 2019 | 10 | Flex./Ext.  Marker on skin  IMU:126(18.85)  OMC:136(17.98)  Marker on sensor  IMU:126(18.85)  OMC:141(20.64)  U/R  Marker on skin  IMU:50.48(9.04)  OMC:51.05(8.44)  Marker on sensor  IMU:50.48(9.04)  OMC:49.91(8.44) | N/A | Flex./Ext.:-9.94(-20.84,0.96)  Radial-ulnar dev.:-0.64(-6.10,4.82) | N/A | N/A | MAD  Flex./Ext.:10.37±4.22  Radial-ulnar dev.:2.42±1.19 |
| Wu, et al. | 2022 | 7 | N/A | Shoulder  Flex./Ext.  Fast:0.92  Slow:0.95  Abd./Add.  Fast:0.87  Slow:0.86  IR/ER  Fast:0.87  Slow:0.89  Elbow  Flex./Ext.  Fast:0.86  Slow:0.90  P-S  Fast:0.90  Slow:0.88 | Shoulder  Flex./Ext.  Fast：7.5  Slow：8.8  Abd./Add.  Fast：5.0  Slow：6.8  IR/ER  Fast：9.0  Slow：8.2  Elbow  Flex./Ext.  Fast12.3  Slow:12.3  P-S  Fast:9.3  Slow:9.5 | Shoulder  Flex./Ext.  Fast:25.9±6.4  Slow:17.9±11.9  Abd./Add.  Fast:-12.6±16.0  Slow:-11.3±13.6  IR/ER  Fast:-13.0±15.9  Slow:-11.5±17.7  Elbow  Flex./Ext.  Fast:27.8±18.8  Slow:27.1±16.1  P-S  Fast:8.3±6.9  Slow:6.6±7.2 | N/A | R2  Shoulder  Flex./Ext.  Fast:0.96  Slow:0.96  Abd./Add.  Fast:0.80  Slow:0.81  IR/ER  Fast:0.79  Slow:0.79  Elbow  Flex./Ext.  Fast:0.95  Slow:0.94  P-S  Fast:0.81  Slow:0.81 |

N/A: Not applicable
